# A New Perspective on Magnetotail Electron and Ion Divergent Flows: MMS Observations

**DOI:** 10.1029/2022JA030514

**Published:** 2022-09-30

**Authors:** T. Motoba, M. I. Sitnov, G. K. Stephens, D. J. Gershman

**Affiliations:** ^1^ The Johns Hopkins University Applied Physics Laboratory Laurel MD USA; ^2^ NASA Goddard Space Flight Center Greenbelt MD USA

**Keywords:** plasma divergent flows, magnetic reconnection, magnetotail plasma watersheds

## Abstract

Fast divergent flows of electrons and ions in the magnetotail plasma sheet are conventionally interpreted as a key reconnection signature caused by the magnetic topology change at the X‐line. Therefore, reversals of the *x*‐component (*V*_*x*⊥_) of the plasma flow perpendicular to the magnetic field must correlate with the sign changes in the north‐south component of the magnetic field (*B*_*z*_). Here we present observations of the flow reversals that take place with no correlated *B*_*z*_ reversals. We report six such events, which were measured with the high‐resolution plasma and fields instruments of the Magnetospheric Multiscale mission. We found that electron flow reversals in the absence of *B*_*z*_ reversals (a) have amplitudes of ∼1,000–2,000 km s^−1^ and durations of a few seconds; (b) are embedded into larger‐scale ion flow reversals with enhanced ion agyrotropy; and (c) compared with conventional reconnection outflows around the electron diffusion regions (EDRs), have less (if ever) pronounced electron agyrotropy, dawnward electron flow amplitude, and electric field strength toward the neutral sheet, although their energy conversion parameters, including the Joule heating rate, are quite substantial. These results suggest that such flow reversals develop in the ion‐demagnetization regions away from electron‐scale current sheets, in particular the EDRs, and yet they play an important role in the energy conversion. These divergent flows are interpreted as precursors of the flow‐driven reconnection onsets provided by the ion tearing or the ballooning/interchange instability.

## Key Points

- Transient electron flow reversals embedded into a more gradual ion flow reversal in the magnetotail occur without magnetic topology change
- Properties of the discovered divergent flows are distinctly different from those of reconnection outflows in the electron diffusion region
- Divergent flows are interpreted as flow‐driven reconnection onsets due to the ion tearing or the ballooning/interchange instability

## 1 Introduction

Magnetic reconnection is a key process in collisionless plasmas that converts magnetic energy into plasma kinetic and thermal energies through a rapid change of magnetic field topology. At the Earth's magnetopause, the reconnection process leads to efficient transport of solar wind energy into the magnetosphere, whereas in the magnetotail, it explosively releases the accumulated magnetic energy and causes global geomagnetic disturbances leading to auroral substorms (Baker et al., 1996; Hones, 1984). The energy conversion and topological changes take place in a very small region surrounding an X‐line where ions or both ions and electrons are decoupled from the magnetic field. These regions are called the diffusion regions. They have a multiscale structure due to different masses and hence dynamics of ions and electrons. A larger ion diffusion region (IDR) with unmagnetized ions usually contains a smaller‐scale electron diffusion region (EDR) where both electrons and ions are unmagnetized.

A distinctive feature of the magnetotail reconnection onset is that corresponding plasma motions are needed to reduce the original finite normal magnetic field for the formation of an X‐line. This is not necessary in case of antiparallel or sheared magnetic field configurations, such as at the magnetopause. Indeed, there is some controversy regarding whether fast plasma divergent flows in the magnetotail are a consequence or a cause of the magnetic topology change. In the former and the most commonly accepted scenario, the magnetic topology first changes because of the slow evolution of the tail induced by the external driving and subsequent microscale tearing instability (e.g., Liu et al., 2014). Therefore, plasma divergent flows arise from the unbalanced magnetic tension of the sharply kinked, newly reconnected field lines around the X‐line.

The latter scenario was originally proposed by Lin and Swift (2002). On the basis of the analysis of their 2‐D global hybrid simulations, they conjectured that the magnetic topology change can be internally driven by the spontaneous generation of plasma divergent flows. The idea of such flows preceding and likely driving the reconnection onset was later elaborated on by Siscoe et al. (2009) and Tanaka et al. (2019) in the analysis of their magnetohydrodynamic (MHD) simulations. These plasma divergent flows are referred to below as plasma “watersheds” (WSs), to distinguish them from reconnection ejecta emanated from an already formed X‐line. Most recently, Sitnov, Motoba, and Swisdak (2021), using fully kinetic 3‐D particle‐in‐cell (PIC) simulations, discovered that the preonset diverging flows have a complex structure with multiple electron WSs being embedded into a larger‐scale ion WS. Although they also provided an example of WS‐like structures from the Magnetospheric Multiscale (MMS) observations in the magnetotail to compare them with the PIC simulation results, the measurements could be mixed with the regimes of electron‐only or guide‐field reconnection (Chen et al., 2019; Phan et al., 2018). Thus, the primary objective of this study is to conduct a detailed multicase investigation of electron and ion WSs in the magnetotail using capabilities of MMS instruments and to determine the distinctive features of WSs.

When a spacecraft is crossing the diffusion regions along the outflow direction in the vicinity of an X‐line, the expected signatures would be a reversal of earthward and tailward convective flows (i.e., *V*_*x*⊥_, perpendicular to the magnetic field in the Geocentric Solar Magnetospheric [GSM] coordinate system) as well as a reversal of the northward component of the magnetic field (*B*_*z*_ in GSM). Indeed, such *V*_*x*⊥_ and *B*_*z*_ (or *V*_*L*⊥_ and *B*_*N*_ in the local coordinate system (so‐called “LMN”), where **L** is in the outflow direction (roughly sunward in the magnetotail), **M** along the X‐line (roughly duskward), and **N** normal to the current sheet (roughly northward)) reversals have been detected in magnetotail observations made by Wind (Øieroset et al., 2001), Geotail (Nagai et al., 2011), Cluster (Runov et al., 2003), Time History of Events and Macroscale Interactions during Substorms (Angelopoulos et al., 2020), and MMS (Torbert et al., 2018). Moreover, the collisionless nature of reconnection has been confirmed by observed signatures of the Hall fields, the bipolar electric field *E*_*z*_ in GSM (or *E*_*N*_ in LMN) toward the neutral plane, and the quadrupolar out‐of‐plane magnetic field *B*_*y*_ in GSM (or *B*_*M*_ in LMN) (e.g., Eastwood, Phan, et al., 2010; Eastwood, Shay et al., 2010).

The unprecedented time and space resolution of the MMS mission allows one to further investigate different regimes of the magnetotail reconnection and its onset details. In this paper, we seek to observationally characterize kinetic field and plasma features of ion and electron WSs in the magnetotail using MMS data, emphasizing how these features contrast with those of conventional reconnection ejecta from the EDR (Torbert et al., 2018) as well as divergent electron flows in electron‐only reconnection regimes (Phan et al., 2018).

The remainder of this study is organized as follows. Section 2 describes the instrumentation and data. Section 3.1 briefly describes the key features of the “benchmark” 11 July 2017 EDR event observed in the magnetotail. In Section 3.2, we first describe the event selection and then present three representative WS events (Events 1–3) by comparing them with the 11 July 2017 EDR event as well as with simulation results of the flow‐driven magnetotail reconnection regimes. In addition, we briefly introduce three other WS events (Events 4–6) that took place in more dipolarized tail regions and compare them with Events 1–3. Section 4 summarizes the results and discusses their implications.

## 2 Data and Instrumentation

MMS (Burch et al., 2016) is composed of four identical spacecraft that were launched in March 2015, to investigate small‐scale reconnection physics in the Earth's magnetosphere, particularly the electron‐scale physics. In this study, we utilize the fast survey and burst mode data of fields and plasma moments acquired from the FIELDS (Torbert et al., 2016) and Fast Plasma Investigation (FPI; Pollock et al., 2016) instruments on board MMS; burst‐mode (128 Hz) magnetic field vector (**B**) data measured by the FIELDS Flux Gate Magnetometer (FGM; Russell et al., 2016); fast survey‐mode (32 Hz) electric field vector (**E**) data measured by the Electric field Double Probes (EDP; Ergun et al., 2016; Lindqvist et al., 2016); and burst‐mode ion (150 ms) and electron (30 ms) moment data at 0.01–30.0 keV/q measured by FPI.

The burst‐mode FPI plasma moment data enable us to directly estimate two important parameters that characterize WSs. One is the current density (**J**): **J** = *qN*_*e*_(**V**_**i**_–**V**_**e**_), where *q* is the elementary charge, *N*_e_ is the electron density (assuming quasi‐neutrality), **V**_**i**_ is the ion bulk velocity, and **V**_**e**_ is the electron bulk velocity. Another is the so‐called *Q*‐parameter (Swisdak, 2016), which is a scalar measure of agyrotropy (non‐gyrotropy) of plasma species that reflects the degree of demagnetization of electrons and ions (mainly protons). The *Q*‐parameter can be calculated from the following equation (its square‐root value is discussed in the literature): *Q*_*α*_^1/2^ = [(*P*_*α*_^2^_12_ + *P*_*α*_^2^_13_ + *P*_*α*_^2^_23_)/(*P*_*α*_^2^_⊥_ + 2 *P*_*α*_^2^_⊥_ *P*_*α*_^2^_||_)]^1/2^, where *α* is species. Here, *Q* ^1/2^ = 0 (*Q* ^1/2^ = 1) means that plasma tends to be gyrotropic (agyrotropic).

## 3 Observations

### 3.1 EDR on 11 July 2017

**Figure 1:**
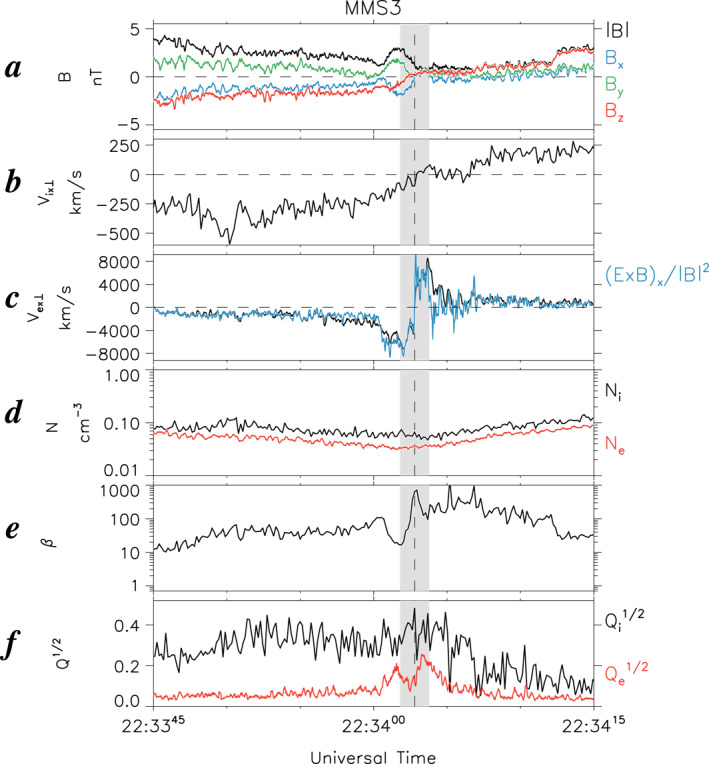
Summary plots of Magnetospheric Multiscale (MMS) 3 observations of the electron diffusion regions event at 2233:45–2234:15 UT on 11 July 2017. (a) Three components and the strength of the magnetic field in Geocentric Solar Magnetospheric [GSM] coordinates (*B*_*x*_, blue; *B*_*y*_, green; *B*_*z*_, red; and |*B*|, black). (b) *x* component of the perpendicular ion velocity (*V*_*ix*⊥_) in GSM coordinates. (c) *x* component of the perpendicular electron velocity (*V*_*ex*⊥_, black) and (E × B)_*x*_/|*B*|^2^ (blue) in GSM coordinates. (d) *N*_*e*_ (red) and *N*_*i*_ (black). (e) Plasma *β*. and (f) Agyrotropy parameter, *Q* ^1/2^; *Q*_*e*_^1/2^ for electrons (red) and *Q*_*i*_^1/2^ for ions (black). Vertical dashed line and gray shading denote the reversal time (*t*_r_) of *V*_*ex*⊥_ at 2234:03 UT and a *t*_r_ ± 1 s range, respectively.

Here let us briefly summarize key features of the magnetotail EDR event on 11 July 2017, which was originally reported by Torbert et al. (2018; hereafter referred to as “T18”), in order to differentiate the plasma WSs presented in Section 3.2 from this classical magnetotail reconnection picture. Figure 1 shows an overview plot of MMS 3 field and plasma measurements for a 30‐s interval of 2233:45–2234:15 UT on 11 July 2017 to characterize the magnetotail EDR. These parameters are displayed in the GSM coordinate system—instead of the LMN coordinate system used in T18—because for this EDR event, the GSM coordinate system is close to the LMN coordinate system (i.e., *x* ∼ *L*, *y* ∼ *M*, and *z* ∼ *N*), as reported by Genestreti et al. (2018) using various methods. We also note that MMS data are shown in the GSM coordinate system throughout the paper, unless otherwise specified.

When MMS 3 encountered the EDR at approximately 2234:03 UT (vertical dashed line), the spacecraft stayed close to the magnetotail central plasma sheet (CPS) at a radial distance of ∼22 *R*_E_, where |*B*| and plasma density (*N*_*e*_ and *N*_*i*_) decreased to a minimum of ∼1 nT (Figure 1a) and ∼0.03–0.05 cm^−3^ (Figure 1d), respectively, whereas the plasma beta (*β*, the ratio of the magnetic and plasma pressures) reached a pronounced peak of ∼800 (Figure 1e).

**Figure 2:**
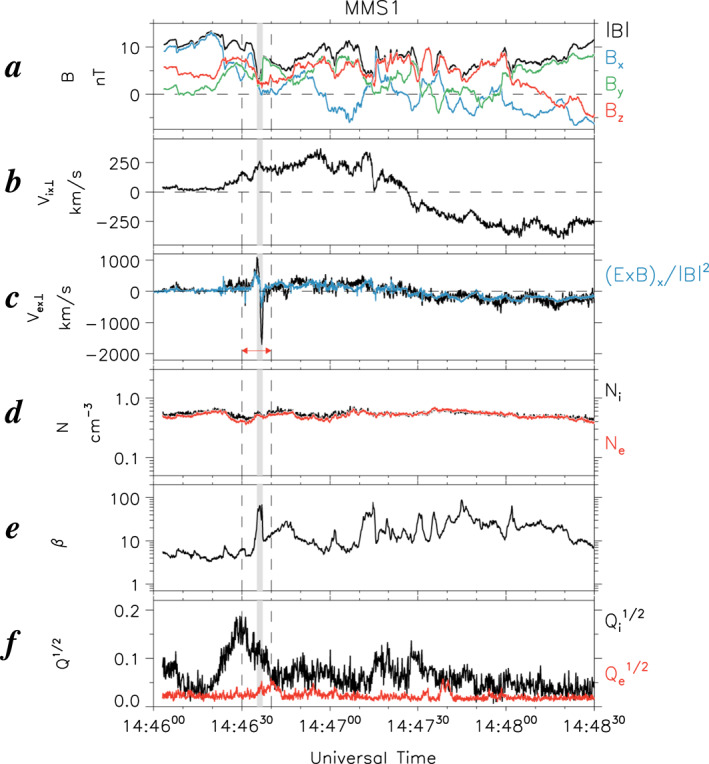
Same format as Figure 1, but for Magnetospheric Multiscale (MMS) 1 observations of Event 1 at 1446:00–1448:30 UT on 2 August 2020. Gray shading denotes the electron watershed.

The EDR properties extracted from the MMS 3 observations, which are key points for the subsequent comparison with WS observations, can be summarized as follows: (a) the *x* components of the perpendicular ion and electron flows, *V*_*ix*⊥_ and *V*_*ex*⊥_, exhibit simultaneous tailward‐to‐earthward reversals in the high*‐β* region (Figures 1b–1e); (b) the flow reversals are accompanied by a negative‐to‐positive sign change in *B*_*z*_ (Figure 1a) and a minimum in plasma density (Figure 1d); (c) the perpendicular electron velocity, *V*_*ex*⊥_, generally follows (*E* × *B*)_*x*_/|*B*|^2^ in the divergent jets (Figure 1c); (d) the dawnward electron velocity component is super‐Alfvénic (*V*_*e*M_ ≈ −15,000 km s^−1^ in LMN; not shown here, but see Figure 2C of T18); (e) electron agyrotropy, *Q*_*e*_^1/2^, exhibits a short‐lived enhancement of ∼0.25 in the vicinity of the *V*_*ex*⊥_ reversal (Figure 1f); and (f) ion agyrotropy, *Q*_*i*_^1/2^, is elevated by ∼0.2–0.4 for a much longer interval (Figure 1f).

Deviations of *V*_*ex*⊥_ from (*E*× *B*)_*x*_/|*B*|^2^ (particularly in the earthward flow region) and *Q*_*e*_^1/2^ enhancement provide strong evidence that electrons are demagnetized in the vicinity of the EDR. Furthermore, different temporal variations of the *Q*_*e*_^1/2^ and *Q*_*i*_^1/2^ enhancements reveal a multiscale structure of the reconnection diffusion region with a smaller‐scale EDR being embedded into a larger‐scale IDR. It is worth noting that **J · E′** = **J ·** (**E** + **V**_**e**_ × **B**), considered as an MHD measure of the Joule dissipation (Birn & Hesse, 2005; Zenitani et al., 2011), becomes positive with a peak value of ∼0.3 nW m^−3^ in the vicinity of the *V*_*ex*⊥_ reversal and that the normal component of the electric field *E*_N_ in LMN (directed toward the neutral plane) has a Hall‐related bipolar signature on the order of several tens of mV m^−1^, as seen in Figures 2H and 2G of T18. Overall, the high‐resolution MMS observations have revealed the theoretically‐predicted properties of the EDR, including the concurrent *V*_*ex*⊥_, *V*_*ix*⊥_, and *B*_z_ reversals, a dawnward super‐Alfvénic electron jet, electron demagnetization (*V*_*ex*⊥_ ≠ (*E* × *B*)_*x*_/|*B*|^2^), *Q*_*e*_^1/2^ enhancement, strong variations of the Joule heating rate **J · E′**, and a strong Hall electric field toward the neutral plane.

In Section 3.2 we will highlight the main properties of electron and ion WSs in the magnetotail by comparing them with the key properties of the T18 EDR event described above.

### 3.2 Plasma WSs

In recent PIC simulations, Sitnov, Motoba, and Swisdak (2021) have provided a number of distinctive features of ion and electron WSs in the magnetotail at the kinetic level of their description. First, electron and ion WSs occur on different spatial and temporal scales: an electron WS has a transient, small‐scale structure compared with a more sustained, larger‐scale ion WS. Second, small‐scale electron WSs are embedded into a larger‐scale ion WS. Third, in the WS region, ions are mostly demagnetized, whereas electrons are mostly magnetized. Fourth, and most importantly, an electron WS is not accompanied by the concurrent magnetic topology change, unlike the EDR. (Note that Sitnov, Motoba, and Swisdak (2021) also found regimes with ion WSs, when the divergent ion flows occur in the absence of the magnetic topology change. However, because MMS provides electron‐scale resolution, these ion‐scale effects are not in the focus of our study). On the basis of such expected properties, we selected six potential WS candidates by visually inspecting the MMS quick‐look burst plots (https://lasp.colorado.edu/mms/sdc/public/) during the 2017–2020 tail phases. All the selected events meet the following criteria: (a) transient reversals of the electron flow perpendicular to the magnetic field (*V*_*ex*⊥_) occur in either the earthward or tailward flow region of a single ion flow reversal (*V*_*ix*⊥_); (b) the transient *V*_*ex*⊥_ reversals occur in the absence of *B*_*z*_ reversals; (c) MMS probes are located in a CPS region of the magnetotail with plasma *β* > 1 (cf. Baumjohann et al., 1989); and (d) the plasma density during WS crossings is greater than 0.1 cm^−3^. The last criterion guarantees a certain level of FPI moment data quality.

In Sections 3.2.1–3.2.3, we first describe each of three representative WS events (Events 1–3) that appear to be most closely similar to the WS simulations by Sitnov, Motoba, and Swisdak (2021) and a manifestation of the flow‐driven reconnection. In Section 3.2.4, we briefly describe three other WSs (Events 4–6) that occurred under stronger *B*_z_ conditions and are interpreted in terms of a mechanism different from that of Events 1–3. Data analysis is largely limited to the MMS 1 observations in the GSM coordinate system. For the interested reader, however, the key parameters at the other available MMS probes and the WS pictures in an LMN coordinate system for selected events are provided in the Supporting Information S1 (SI).

#### 3.2.1 2 August 2020 Event: Event 1

The first WS event, Event 1, took place on 2 August 2020 when the MMS tetrahedron was located at (*X*, *Y*, and *Z*) = (−28.0, −1.8, 4.0) *R*_E_ in GSM coordinates, with an interspacecraft separation of ∼40 ± 5 km. Event 1 occurred under geomagnetically active conditions when the *z* component of the interplanetary magnetic field (IMF *B*_*z*_) ranged from −5 to −10 nT for several hours (Figure S1 in Supporting Information S1), and it was the only event in which *B*_*z*_ decreased to ∼2 nT, in contrast to the other five events where *B*_*z*_ > 5 nT.

Figure 2 displays MMS 1 observations of Event 1 at 1446:00–1448:30 UT. At ∼1446:36 UT the *x* component of the perpendicular electron velocity (*V*_*ex*⊥_) made a transient, earthward‐to‐tailward reversal (Figure 2c). The *V*_*ex*⊥_ reversal time, highlighted by gray shading, is hereinafter referred to as *t*_*r*_. During the transient reversal, the *V*_*ex*⊥_ value varied from approximately 1,000 to −1,500 km s^−1^. These peak values are greater than the local ion Alfvén speed (∼120–300 km s^−1^) based on |*B*| = 4–10 nT and *N*_*i*_ = 0.5 cm^−3^. Interestingly, the *x* component of the perpendicular ion velocity (*V*_*ix*⊥_, Figure 2b) did not respond to the transient *V*_*ex*⊥_ reversal at all, indicating that electrons and ions were decoupled. The transient flow reversal of electrons was embedded in a more gradual earthward‐to‐tailward flow reversal of ions with |*V*_*ix*⊥_| ∼ 300 km s^−1^, which took place at ∼1447:25 UT. Hereafter these electron and ion divergent flows are referred to as electron and ion WSs, respectively.

Within the transient electron WS, the magnetic field strength, |*B*| (Figure 2a), decreased from ∼10 to ∼4 nT, whereas the plasma density (*N*_i_ and *N*_e_, Figure 2d) slightly increased from 0.4 to 0.5 cm^−3^. As a result, the plasma *β* value sharply jumped from ∼5 up to ∼60 (Figure 2e). We also found that the electron WS appeared within an extended interval of elevated ion agyrotropy, *Q*_i_^1/2^ ∼ 0.18 (Figure 2f). Both results indicate that the electron WS was located in a CPS region of the magnetotail where ions were unmagnetized.

On a larger scale, the ion WS at ∼1447:25 UT occurred under positive values of *B*_*z*_ ∼ 5–10 nT, as predicted for WS simulations. Approximately 50 s later, *B*_*z*_ reversal took place, suggesting that the ion‐scale reversal could also be interpreted as an IDR (e.g., Rogers et al., 2019). With the present MMS probe separation, however, it is impossible to judge whether the ion flow drove the topology change or the latter took place before the ion flow reversal but in a different region in space, which the MMS spacecraft detected later.

**Figure 3:**
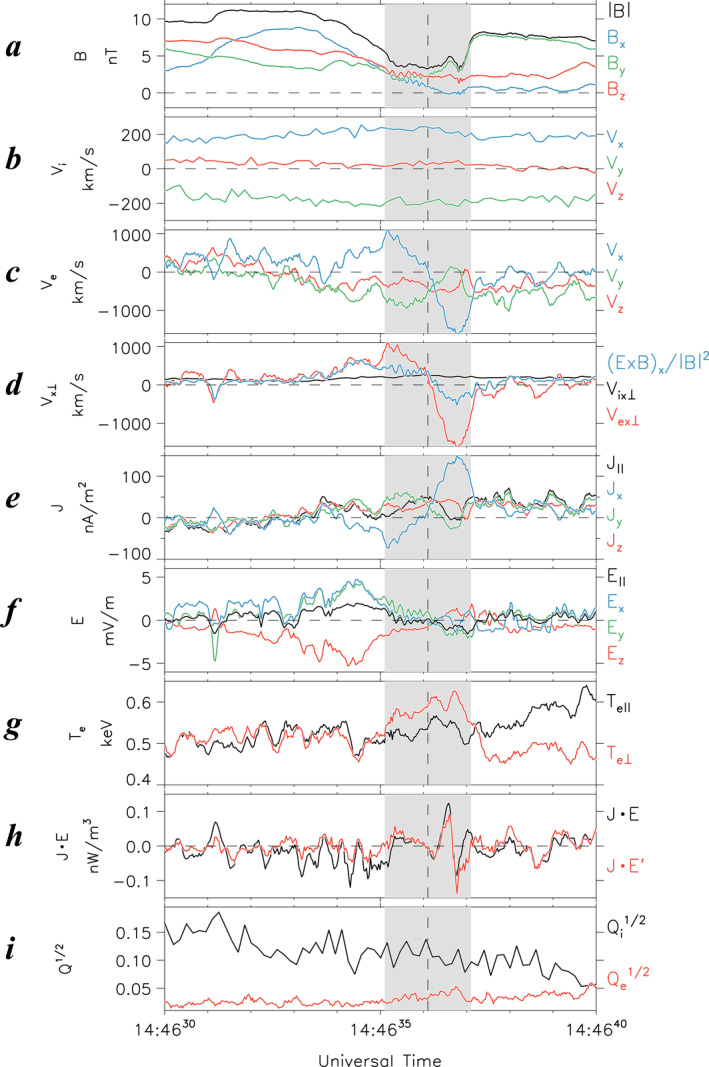
Zoomed‐in plots of Event 1 for a 10‐s interval of 1446:30–1446:40 UT on 2 August 2020, which appears between dashed lines in Figure 2. (a) Magnetic field in GSM coordinates (*B*_*x*_, blue; *B*_*y*_, green; *B*_*z*_, red; and |*B*|, black). (b) Ion velocity in GSM coordinates (*V*_*ix*_, blue; *V*_*iy*_, green; and *V*_*iz*_, red). (c) Electron velocity in GSM coordinates (*V*_*ex*_, blue; *V*_*ey*_, green; and *V*_*ez*_, red). (d) *V*_*ex*⊥_ (red), *V*_*ix*⊥_ (black), and (**E** × **B**)_*x*_/|*B*|^2^ (blue) in GSM coordinates. (e) Current density in GSM coordinates (*J*_*x*_, blue; *J*_*y*_, green; *J*_*z*_, red; and *J*_||_, black). (f) Electric field in GSM coordinates (*E*_*x*_, blue; *E*_*y*_, green; *E*_*z*_, red; and *E*_||_, black). (g) *T*_e⊥_ (red) and *T*_e||_ (black). (h) ***J* **·** *E*** (black) and ***J* **·** *E***_***e***_' (red). (i) Ion and electron agyrotropies, *Q*_*i*_^1/2^ (black) and *Q*_*e*_^1/2^ (red). Dashed line and gray shading denote the reversal time (*t*_r_ = ∼1446:36.1 UT) of *V*_*ex*⊥_ and a *t*_r_ ± 1 s range, respectively.

To further analyze the electron WS, Figure 3 presents a zoomed‐in view of the field and plasma data during the 10‐s interval of 1446:30–1446:40 UT, marked by two dashed vertical lines in Figure 2. Shown from top to bottom are **B** (Figure 3a); **V**_i_ (Figure 3b); **V**_e_ (Figure 3c); *V*_*ex*⊥_, *V*_*ix*⊥_ and (*E* × *B*)_*x*_/|*B*|^2^ (Figure 3d); **J** (Figure 3e); **E** (Figure 3f); *T*_*e*⊥_ and *T*_*e*||_ (Figure 3g); **J** · **E** and **J** · **E**' (Figure 3g); and ion and electron agyrotropy, *Q*_i_^1/2^ and *Q*_e_^1/2^ (Figure 3i). A vertical dashed line is drawn at *t*_r_ = 1446:36 UT, and the gray shading represents an interval of *t*_r_ ± 1 s. We also provide in Figure S2 of Supporting Information S1 the same parameters displayed in an LMN coordinate system. Similar to the T18 EDR event, the normal component is almost in the *z* direction in the GSM coordinate system.

It is important to note here that the interpretation of the Joule heating rate must be used with an extreme caution. As already shown in Equation 8 of Zenitani et al. (2011), in quasi‐neutral plasmas, the parameter **J** · **E′** cannot distinguish between ion and electron species. Therefore, it cannot be used as a measure of the kinetic (Landau) dissipation, which is distinctly different for different plasma species. Thus, in the analysis of collisionless dissipation in magnetic reconnection, **J** · **E′** must be replaced by the kinetic analogs based on the pressure‐strain interaction (Bandyopadhyay et al., 2021; Sitnov et al., 2018, 2021b; Yang et al., 2017). At the same time, the MMS tail observations pose another challenge because of an insufficient probe spacing, which may not match the time resolution (Sitnov, Stephens, et al., 2021). This is why below we have to limit our analysis by the parameters **J** · **E** and **J** · **E′**.

Similar to the T18 EDR event (Figure 1a), this electron WS occurred at a minimum |*B*| that rapidly dropped to ∼4 nT (Figure 3a). However, we emphasize that most other properties of this WS are drastically different from the T18 EDR event. First, the sign of the normal magnetic field, *B*_*z*_, remained unchanged (Figures 3a and 3d), suggesting the preserved tail topology during this event.

Second, the electron flow velocity reversal was not accompanied by a comparable peak in the dawnward electron velocity (|*V*_*ey*_| ≤ |*V*_*ex*_|, Figure 3c) or the duskward current density (|*J*_*y*_| ≤ |*J*_*x*_|, Figure 3e). This result can be contrasted with Figures 2C and 2E in T18, showing dawnward super‐Alfvénic electron jets with |*V*_*eM*_| ∼15,000 km s^−1^ > |*V*_*eL*_| and the corresponding strong duskward current density with |*J*_*M*_| ∼ 100 nA m^−2^ > |*J*_*L*_|.

Third, the electric field *E*_*z*_ normal to the neutral plane was relatively weak (|*E*_*z*_| < 2 mV m^−1^, Figure 3f), unlike Figure 2g in T18 indicating strong bipolar *E*_*N*_, pointing toward the center of the current sheet (the Hall electric field) with |*E*_*N*_| ∼ 30 mV m^−1^. This result, together with the second result, suggests that the electron WS region was away from electron‐scale thin current sheets.

Fourth, the quantitative discrepancy between |*V*_*ex*⊥_| and |(*E* × *B*)_*x*_/|*B*|^2^| in the electron WS region (Figure 3d) was larger, compared with the T18 EDR event (Figure 1c). Such strong deviations of magnetized electron flows (red curve in Figure 3i) from their frozen‐in motion can be explained by their Hall interaction with unmagnetized ions (black line in Figure 3i) as is the case in the ion‐tearing mode (e.g., Sitnov & Swisdak, 2011).

At the same time, whereas the energy conversion rate **J** · **E** and the Joule heating rate **J** · **E′** were nearly zero at *t*_r_ (Figure 3h), ∼1 s later, they had substantial positive and negative excursions with amplitudes of ∼0.1 nW m^−3^ comparable to those for the T18 EDR event (**J** · **E′** ∼ 0.3 nW m^−3^). This suggests that, like the EDR, this electron WS was a site of the strong energy conversion, consistent with the ion‐tearing mechanism (Schindler, 1974; Sitnov et al., 2013; Sitnov & Swisdak, 2011).

To make sure that the obtained WS picture is consistent with other MMS probe observations, we provide in Figure S3 of Supporting Information S1 an analog of Figures 3a–3c using data from all available MMS probes. Note that the FPI electron and ion moment data at MMS 2 and the FPI electron moment data at MMS 4 are not available in the required burst mode during Event 1. It is evident from Figure S3 in Supporting Information S1 that the magnetic field and bulk flow velocity data at all available probes are generally consistent with each other. At the same time, such a similar picture makes it hard to clarify the global reconnection geometry because of much smaller probe separation than missions like Cluster (Eastwood et al., 2005).

**Figure 4:**
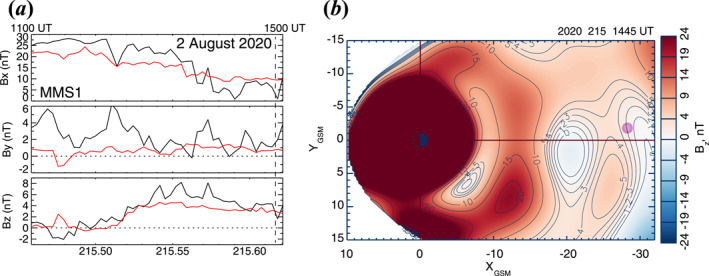
Data mining (DM) reconstructions of the magnetic field during Event 1 on 2 August 2020 (day of year: 215). (a) Comparison between the DM‐reconstructed and observed magnetic field at Magnetospheric Multiscale (MMS) 1 for the interval of 1100–1500 UT. Dashed line is drawn at 1445 UT when MMS 1 was closest to the watershed. (b) A snapshot of *B*_*z*_ distribution in the equatorial plane at 1445 UT. Pink circle denotes the MMS 1 location.

To further clarify the global context of Event 1, we used global reconstructions of the magnetic field that have become possible because of the application of the data mining (DM) technique to historical archives of multi‐mission magnetometer data (Sitnov et al., 2019; Stephens et al., 2019). The DM provides the geomagnetic field reconstructions with a time sampling of 5 min, and the reconstructions are quantified by the SuperMAG geomagnetic indices, SMR and SML (equivalent to Sym‐H and AL), their time derivatives, and the solar wind input parameter *V*_sw_ · *B*_*s*_^IMF^ (*V*_sw_ is the solar wind velocity and *B*_*s*_^IMF^ is equivalent to IMF *B*_z_). The DM outputs for Event 1 are shown in Figure 4.

Figure 4a presents comparison of 5‐min averaged magnetic field vectors observed at MMS 1 (black curves) with the DM outputs (red curves) for the interval of 1100–1500 UT. The DM outputs represent magnetic field vectors reconstructed along the MMS 1 orbit. The moment closest to the WS observations at MMS (1445 UT) is marked by the dashed vertical line. Throughout the interval, the DM output generally follows the observed magnetic field variations, although there are some exceptions, particularly for the *x*‐ and *y*‐components. Such a good agreement between the observed and reconstructed magnetic fields at MMS 1 guarantees a certain level of DM output reliability.

Figure 4b shows the distribution of *B*_*z*_ in the equatorial plane of the magnetosphere at 1445 UT. The pink circle indicates the MMS 1 location. The DM results suggest that the observed electron and ion WSs took place just earthward of a global *B*_*z*_‐reversal region at about −30 *R*_E_, consistent with the PIC simulation picture demonstrated by Sitnov, Stephens, et al. (2021).

#### 3.2.2 3 July 2017 Event: Event 2

The second WS event, Event 2, took place at 0526:30–0527:15 UT on 3 July 2017 when the MMS tetrahedron was located at a downtail radial distance of ∼18 *R*_E_, (*X*, *Y*, and *Z*) = (−17.6, 3.3, 1.7) *R*_E_ in GSM. The average interspacecraft separation was ∼25 km during this interval. This event, close to a series of dipolarization fronts (DFs), was originally reported by Chen et al. (2019), who focused on the guide‐field reconnection near EDRs. On the other hand, based on comparison with 3‐D PIC simulations, Sitnov, Motoba, and Swisdak (2021) suggested that electron and ion WSs also manifested during this interval. Here we provide a more detailed analysis of the WS signatures using the MMS observations. Note that not all data from MMS 4 instruments are available for Event 2. Below we present field and plasma observations obtained from MMS 1 in GSM coordinates, but these observations in an LMN coordinate system and comparisons among all three probes are provided in Figures S5 and S6 of Supporting Information S1, respectively.

**Figure 5:**
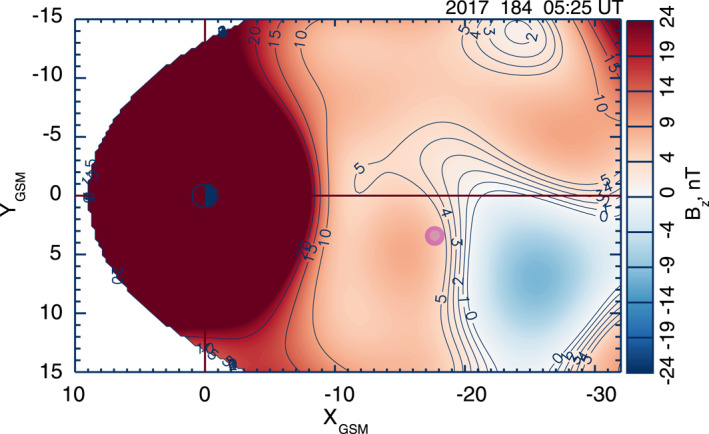
Same as Figure 4b but for Event 2 at 0525 UT on 3 July 2017 (day of year: 184). Pink circle denotes the Magnetospheric Multiscale location.

Whereas Event two occurred under relatively quiet geomagnetic conditions just before a small substorm initiated at ∼0540 UT (Figure S4 in Supporting Information S1), the DM‐based reconstruction at 0,525 UT (Figure 5) indicates that this WS event was located in a region of relatively small *B*_z_ value earthward of a global X‐line (*B*_*z*_‐reversal region) at the time of interest, similar to the WS simulation picture (Sitnov, Motoba, & Swisdak, 2021).

**Figure 6:**
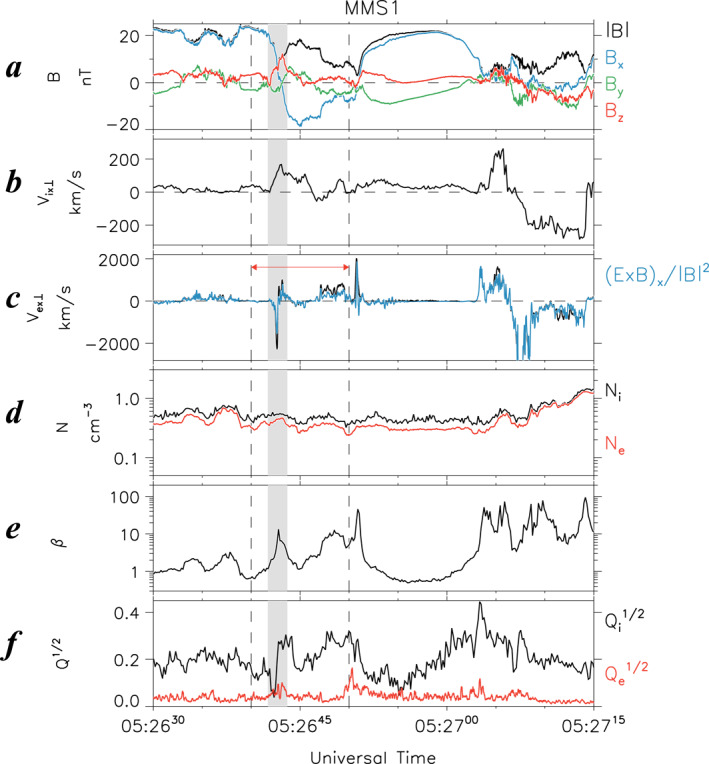
Same format as Figure 1, but for Magnetospheric Multiscale (MMS) 1 observations of Event 2 at 0526:30–0527:15 UT on 3 July 2017. Gray shading denotes the electron watershed.

Figure 6 shows an overview of Event 2 observed by MMS 1 at 0526:30–0527:15 UT. It is evident from Figure 6a that throughout this interval, the MMS 1 spacecraft encountered a short‐period flapping of the magnetotail current sheet characterized by strong *B*_*x*_ oscillations with the amplitudes of ∼10–20 nT and the periods of ∼10–15 s. At ∼0526:42 UT (marked by gray shading), immediately before the first neutral sheet crossing (*B*_*x*_ = 0) due to the magnetotail flapping, MMS 1 observed a transient, tailward‐to‐earthward electron flow reversal in *V*_*ex*⊥_ (i.e., electron WS) from approxymately −2,000 km s^−1^ to ∼500–1,000 km s^−1^ (Figure 6c). The electron WS, appearing in an earthward ion flow (*V*_*ix*⊥_, Figure 6b), was not accompanied by a sign reversal of *B*_z_ (Figure 6a). The *B*_z_ field and plasma density near the electron WS were elevated from ∼0 nT up to ∼12 nT (Figure 6a) and from ∼0.3 up to ∼0.5 cm^−3^ (Figure 6d), respectively. Inside the electron WS region, the plasma *β* value (Figure 6e) was greater than 10. According to Figure 6f, ions around the electron WS were unmagnetized/agyrotropic, whereas electrons were magnetized.

**Figure 7:**
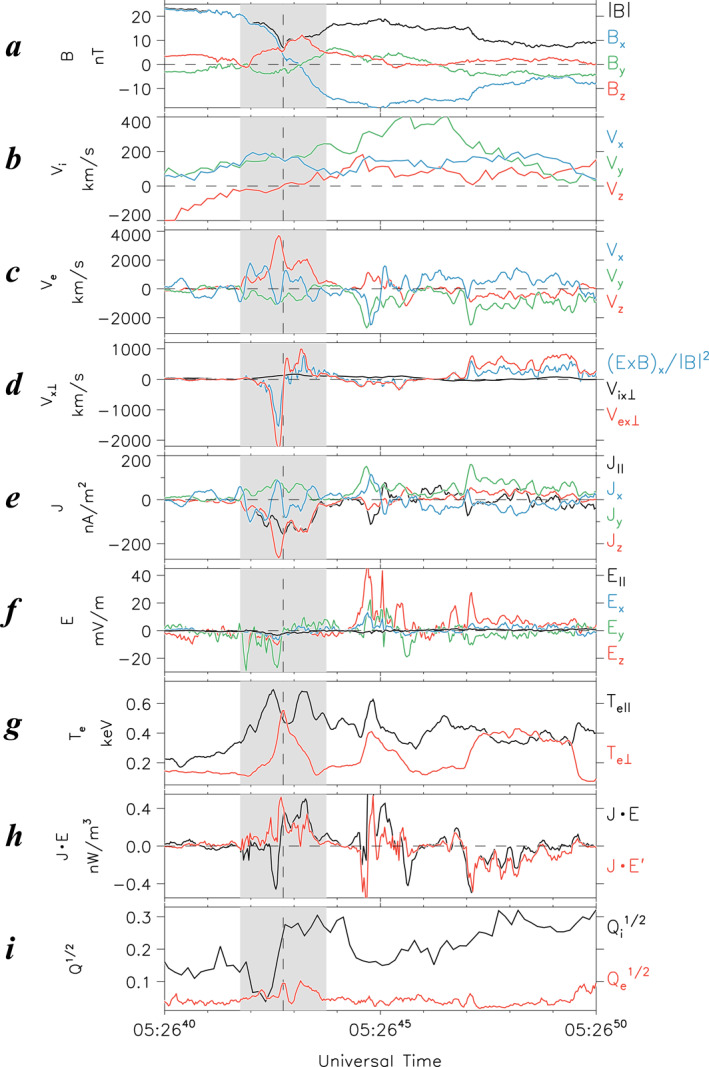
Same format as Figure 3, but for Event 2 at 0526:40–0526:50 UT on 3 July 2017, which appears between dashed lines in Figure 6. Dashed line and gray shading denote the reversal time (*t*_r_ = ∼0526:42.7 UT) of *V*_*ex*⊥_ and a *t*_r_ ± 1 s range, respectively.

Figure 7 presents a zoomed‐in plot of the MMS 1 observations at 0526:40–0526:50 UT to characterize fields and plasma signatures in the vicinity of the electron WS. Such detailed field and plasma signatures confirm that the divergent electron flows at ∼0526:42 UT occurred concurrently with positive *B*_z_ values (Figure 7a), earthward ion flows (Figure 7b), small dawnward bulk electron flow (|*V*_ey_| ≤ 1,000 km s^−1^, Figure 6c), and small electric fields (particularly *E*_z_ in Figure 7f). Similar to Event 1, these features indicate that this electron WS occurred away from any electron‐scale thin current sheet.

In the vicinity of this electron WS, one may notice that the energy conversion and the Joule heating rate had large values (up to 0.4 nW m^−3^, Figure 7h) comparable to those in the T18 EDR, and that the parallel and perpendicular electron temperatures were locally enhanced (Figure 7g). The results suggest that this electron WS was a site of the significant energy conversion and electron heating. Moreover, the electron anisotropy had a stronger enhancement compared with Event 1.

Another interesting feature of Event 2 is that the electron WS had strong, localized negative and positive excursions in *E*_*y*_ (|*E*_*y*_| > 20 mV m^−1^, Figure 7f), corresponding to tailward and earthward electron convective motions in *V*_*ex*⊥_. These motions are expected as a signature of the flux starvation effect associated with WSs in the wakes of DFs (Pritchett, 2015; Sitnov, Motoba, & Swisdak, 2021).

#### 3.2.3 3 August 2020 Event: Event 3

**Figure 8:**
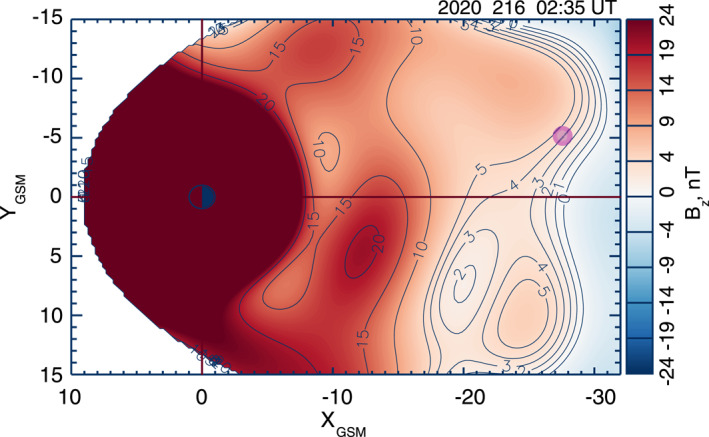
Same as Figure 4b but for Event 3 at 0235 UT on 3 August 2020 (day of year: 216). Pink circle denotes the Magnetospheric Multiscale location.

The third WS event on 3 August 2020, Event 3, took place in an active magnetotail between two nearby substorm activations (Figure S7 in Supporting Information S1). During Event three, the MMS tetrahedron was located at ∼28 *R*_E_ downtail, (*X*, *Y*, and *Z*) = (−27.2, −5.1, 1.4) *R*_E_ in GSM. The separation of the MMS tetrahedron was ∼35–40 km. The DM‐based reconstruction at 0235 UT (Figure 8) indicates that Event 3 occurred in a region of small *B*_z_ value (∼4 nT) earthward of a global X‐line at the time of interest.

**Figure 9:**
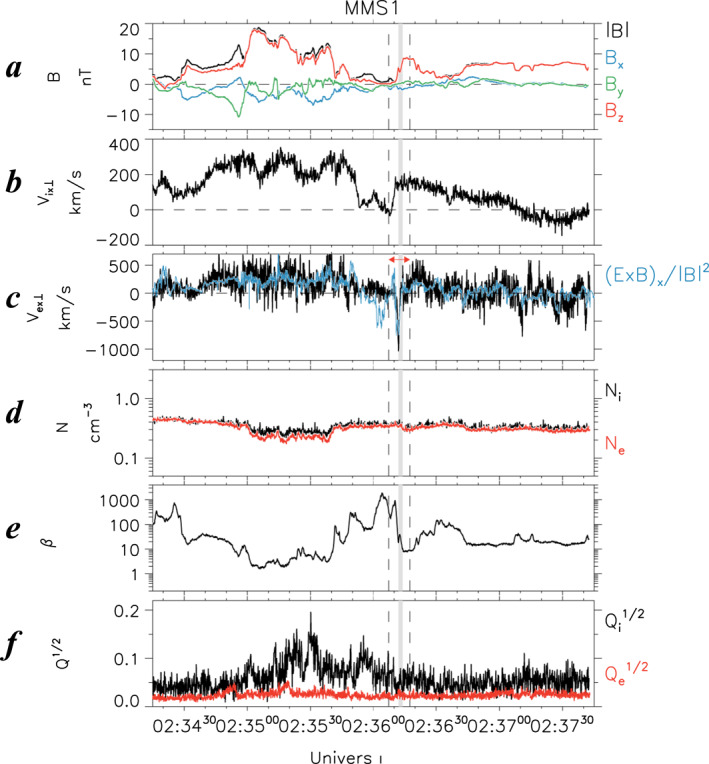
Same format as Figure 1, but for Magnetospheric Multiscale (MMS) 1 observations of Event 3 at 0234:15–0237:45 UT on 3 August 2020. Gray shading denotes the electron watershed.

Figure 9 presents an overview of Event 3 observed by MMS 1 at 0234:15–0237:45 UT. As evident from Figures 9a and 9b, the interval started with a strong DF (*B*_*z*_ (or |*B*|) enhancement by ∼18 nT) that occurred under fast earthward ion flow conditions with a speed of ∼300 km s^−1^. After the strong DF passed, MMS 1 encountered a minimum |*B*| (∼0 nT) at ∼02:36:05 UT and then a small *B*_*z*_ enhancement with the amplitude <10 nT. In the course of the small *B*_*z*_ enhancement, a transient electron flow reversal from approximately −1,000 to ∼500 km s^−1^ emerged in the magnetotail CPS where *N* ∼ 0.3 cm^−3^ (Figure 9d), *β* > 10 (Figure 9e), and *Q*_i_^1/2^ ∼ 0.05 and *Q*_e_^1/2^ ∼ 0.02 (Figure 9f). An interesting feature of this case is that under stable positive *B*_z_ (∼5 nT) conditions after the electron WS at ∼0236:13 UT, the ion velocity *V*_*ix*⊥_ reversed its sign from positive to negative, which may be interpreted as a signature of ion WS. This reversal is also consistent with the WS simulation picture (Sitnov, Motoba, & Swisdak, 2021).

We note that the strong variations of the *B*_z_ field seen in Figure 9a made applying the LMN coordinate system to Event 3 difficult. This is because the *L* component in LMN would be close to the *x* direction in GSM, as seen in earlier studies (Marshall et al., 2020). This might suggest the filamentation of the original tail current sheet and even its strong bending, which would distort the global picture for this WS predicted by DM reconstruction (Figure 8). Thus, the subsequent analysis of Event 3 is limited to the GSM system.

**Figure 10:**
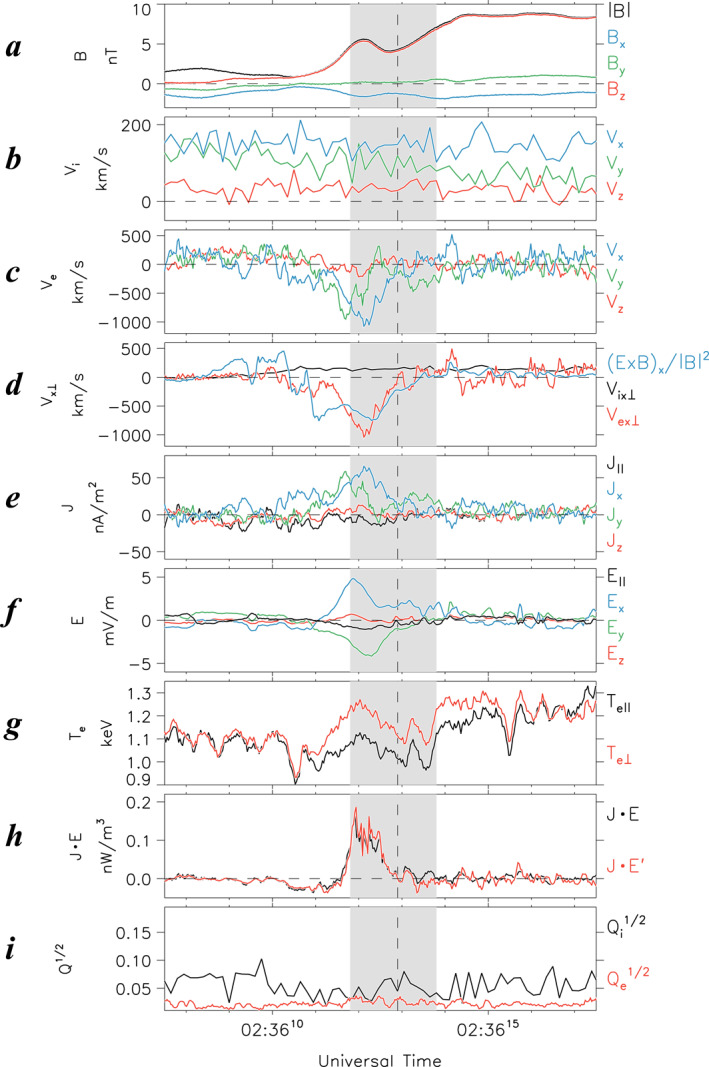
Same format as Figure 3, but for Event 3 at 0236:07.5–0236:17.5 UT on 3 August 2020, which appears between dashed lines in Figure 9. Dashed line and gray shading denote the reversal time (*t*_r_ = ∼0236:12.9 UT) of *V*_*ex*⊥_ and a *t*_r_ ± 1 s range, respectively.

Figure 10 shows a zoomed‐in view of Event 3 for a 10‐s interval of 0236:07.5–0236:17.5 UT with focus on the electron WS at ∼0236:13 UT. It is clear that the electron WS properties share commonalities with those for Events 1 and 2: (a) weak duskward electron velocity enhancement, |*V*_ey_| < 1,000 km s^−1^ (Figure 10c), compared with T18; (b) weak duskward current density, |*J*_y_| ≤ 50 nA m^−2^ (Figure 10e), compared with ∼100 nA m^−2^ in T18; and (c) a small Hall electric field, |*E*_z_| < 5 mV m^−1^ (Figure 10f), compared with ∼30 mV m^−1^ in T18. Consistent with both Events 1 and 2, the energy conversion and Joule heating rate values are substantial up to ∼0.2 nW m^−3^. This result suggests that, similar to EDRs, the observed electron WS is a localized site of the strong energy conversion associated with either plasma instabilities and/or magnetic reconnection. We have also confirmed the consistency of these WS properties at all available MMS probes (Figure S8 in Supporting Information S1).

#### 3.2.4 Other Three Events: Events 4–6

**Figure 11:**
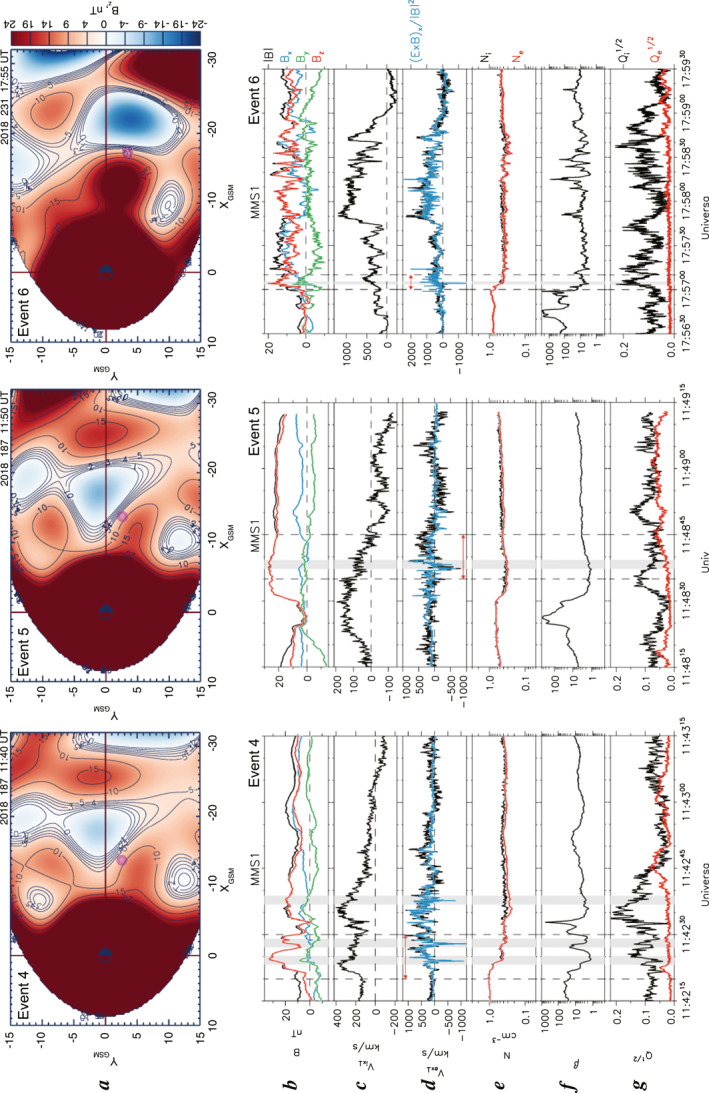
DM reconstructions and Magnetospheric Multiscale (MMS) 1 observations of other three watershed (WS) events: Event 4 (left) and Event 5 (center) on 6 July 2018 and Event 6 (right) on 19 August 2018. (a) Same format as Figure 4b but for Event 4 (1140 UT), Event 5 (1150 UT), and Event 6 (1755 UT). (b–g) Same format as Figures 1a–1f but for Events 4–6. Gray shadings denote the electron WSs.

Here we briefly describe other three WS events, Events 4–6, that took place at 1142:15–1143:15 UT and at 1148:15–1149:15 UT on 6 July 2018 and at 1756:30–1759:30 UT on 19 August 2018. During Events 4 and 5, the MMS spacecraft were located at (X, Y, and Z) = (−13.2, 2.9, 3.6) *R*_E_ in GSM, whereas they were located at (X, Y, and Z) = (−16.9, 3.2, 3.9) *R*_E_ in GSM during Event 6. Figure 11 presents the DM reconstructions and MMS 1 observations of Events 4–6 (left, Event 4; center, Event 5; right, Event 6). Further detailed descriptions and data analyses for each event are provided in Supporting Information S1.

In contrast to Events 1–3, where the WSs were located in regions of relatively small *B*_z_ values in the stretched tail configuration and similar to the WS simulation picture by Sitnov, Motoba, and Swisdak (2021), Events 4–6 were located in strongly dipolarized regions where *B*_z_ values remained large (>15 nT, Figure 11b). The MMS observations are consistent with their global DM reconstructions (Figure 11a), indicating that Events 4–6, being also located close to the global X‐lines, appeared in the regions/times of the magnetotail dipolarization. Events 4–6 exhibit rapid tailward‐then‐earthward motions of the dipolarized flux tubes with magnetized electrons against the background of the earthward ion flows that changed to tailward flows under large and positive *B*_z_ field conditions. Whereas these motions fit the formal definition of plasma WSs, the mechanism for Events four–six is likely different from the pre‐reconnection processes described in Sitnov, Motoba, and Swisdak (2021), as will be discussed below.

## 4 Summary and Discussion

In this study we have investigated the MMS observations of magnetotail plasma divergent flows that change their direction from earthward to tailward or vice versa at radial distances from −13 to −30 *R*_E_. The key features of the observed electron and ion flow reversals in the magnetotail can be summarized as follows:

1. Transient electron divergent flows of ∼1,000–2,000 km s^−1^ in *V*_*ex*⊥_ (electron WSs) are embedded into a single, more gradually changing ion divergent flow in *V*_*ix*⊥_ (ion WS). In electron WSs, magnetized electrons are decoupled from demagnetized.
2. Electron WSs are not accompanied by concurrent sign changes of the northward magnetic field *B*_*z*_ (>4 nT), in strong contrast with the T18 EDR. This indicates that the electron WSs are not associated with any rapid change in magnetic topology.
3. Electron WSs are not accompanied by any significant dawnward electron flows and the corresponding duskward current density enhancements, unlike the T18 EDR. In particular, the duskward electron flows are smaller than the divergent electron flows (|*V*_*ey*_| ≤ |*V*_*ex*_|).
4. The Hall electric field, *E*_*z*_, is rather weak inside the electron WS, compared with strong electric fields directed to the electron‐scale and electron‐dominated current sheet of the T18 EDR. The result indicates that electron WSs are formed away from EDRs.
5. The energy conversion and Joule heating rate in electron WSs are comparable with those in the EDR, although their specific distributions may differ. This is generally consistent with that fact that the relative difference between |*V*_*ex*⊥_| and |(*E* × *B*)_*x*_/|*B*|^2^| is significant in most (not all) of the presented electron WSs. Thus, it is suggested that WSs are active regions of the energy exchange between the electromagnetic field and different plasma species and probably associated with the corresponding multiscale plasma instabilities and magnetic reconnection.

We confirmed for Events 1–6 that these conclusions are consistent for all available MMS probes. For Events 1 and 2 we also confirmed that the results in the original GSM coordinate system are consistent with those in the LMN coordinate system. On the other hand, applications of the GSM‐to‐LMN coordinate transformation to the other four events (Events 3–6) that were detected close to DFs or even just dominant *B*_z_ regions might be misleading in the original context of the divergent tailward‐to‐earthward plasma flows because the results would reflect the DF features. At the same time, the global context of the observed WS events had been clarified by DM reconstruction of the global magnetospheric magnetic field in the equatorial plane (Figures 4, 5, 8, and 11a).

Our analysis shows that the observed divergent flows are drastically different from reconnection outflows from EDRs. Instead, based on their distinctive features (key features 1–5 above), we interpret them as divergent plasma flows preceding the magnetic topology change. The corresponding concept was originally introduced by Lin and Swift (2002) and further developed by Siscoe et al. (2009) and Tanaka et al. (2019). Most recently, it was elaborated as a multiscale and multispecies phenomenon by Sitnov, Motoba, and Swisdak (2021), who reproduced electron and ion WSs using 3‐D PIC simulations and introduced terms “plasma watersheds” and “electron/ion watersheds” to distinguish those pre‐reconnection phenomena from more conventional reconnection outflows, as well as to separate WSs of different plasma species.

In addition to the local MMS observations on electron scales, we have also provided a global picture of the magnetotail reconnection by mining the historical space magnetometer data (Sitnov et al., 2019; Sitnov, Stephens et al., 2021; Stephens et al., 2019). According to this picture, shown in Figures 4, 5, 8 and 11, the MMS spacecraft observing the ion and electron WSs were consistently located just earthward of the global reconnection X‐lines. Note here that the very high fidelity of the X‐line reconstruction using this DM method has recently been provided by Stephens et al. (2022). This finding strongly suggests that all the observed WSs are indeed associated with the magnetotail reconnection processes. It also suggests the possibility that the WSs preceded the subsequent or secondary reconnection processes earthward of the global X‐lines.

Because the reported electron WSs do not coincide with similar ion WSs and occur in earthward and rather weakly disturbed convective ion flows, one may attempt to interpret the *V*_*ex*⊥_ reversal jets in terms of electron‐only reconnection regimes, similar to those observed by MMS in the turbulent magnetosheath (Phan et al., 2018). Indeed, the electron‐only reconnection is maintained by super‐Alfvénic electron flow reversals accompanied by the reversals of the magnetic field normal to the current sheet plane in the absence of the corresponding ion outflows. However, the electron WSs are not accompanied by any normal magnetic field reversals. Furthermore, the electron WSs are often embedded in a larger‐scale ion WS, whereas the electron‐only reconnection is not accompanied by any ion reversal jets. Thus, the observed electron WSs cannot be explained in terms of the electron‐only reconnection.

At the same time, the formation of WSs as a result of the ion tearing instability and/or the subsequent DF dynamics suggested by 3‐D PIC simulations (Sitnov, Stephens, et al., 2021) is not the only possible WS‐formation mechanism. Another possible mechanism is kinetic ballooning/interchange instability (BICI), which can also provide plasma divergent flows (Panov, Nakamura, et al., 2012). Indeed, if the instability region passes transversely across the spacecraft in the *y* direction, it might observe similar *V*_*ex*⊥_ reversals due to the azimuthal structure of the instability (Figure 11 in Panov, Nakamura, et al., 2012). The BICI interpretation may be particularly relevant to Events 4–6 because they occurred in strongly dipolarized regions with large *B*_*z*_ values. However, there are also important distinctions between WSs and conventional BICI perturbations. First, the fast flows in BICI are often field‐aligned (Panov, Sergeev, et al., 2012; Pritchett et al., 2014). Second, in both simulations (Pritchett & Coroniti, 2010) and observations (Panov, Nakamura, et al., 2012; Panov, Sergeev et al., 2012), BICI‐related divergent flows appear as a sawtooth‐like structure, whereas WSs exhibit solitary perturbations with comparable field‐aligned and perpendicular flows or with dominant perpendicular flows. Third, in BICI, the electron velocity oscillations are accompanied by similar *B*_*x*_ and *B*_*z*_ perturbations (e.g., Figure 10 in Panov, Nakamura, et al., 2012) even if they do not change the magnetic topology.

On the other hand, the onset of reconnection caused by BICI waves discovered in some BICI simulations (Pritchett & Coroniti, 2013) and observations (Panov et al., 2020) may resemble the WS onset mechanism (Sitnov, Motoba, & Swisdak, 2021) with the main difference being that the specific non‐reconnection instability generates the divergent plasma motions that cause the topology change. In the case of BICI, these are buoyant motions of plasma with different electron and ion motions due to the lower‐hybrid drift effects (Huba et al., 1977). In the case of WS, this is the ion‐tearing instability (Bessho & Bhattacharjee, 2014; Pritchett, 2015; Schindler, 1974; Sitnov et al., 2013, Sitnov, Motoba, & Swisdak, 2021; Sitnov & Schindler, 2010), which involves different motions of unmagnetized ions and magnetized electrons. Further separation of these mechanisms requires comprehensive multiprobe investigations with the probe arrays extended both along and across the tail current sheet (e.g., Kepko, 2018).

The WS regime belongs to a broader class of flow driven reconnection processes, such as the magnetic shear instability in accretion disks (Hawley & Balbus, 1992), kink instability of the flux ropes in the solar corona (Markidis et al., 2014; Török & Kliem, 2005), reconnection driven by the electron dynamics in laser‐produced plasma (Kuramitsu et al., 2018) and by the Kelvin‐Helmholtz instability at the magnetopause (Ma et al., 2014; Nakamura et al., 2017), and the shock‐driven reconnection (Bessho et al., 2022), which is gaining more and more attention in reconnection physics (Ji et al., 2022). The special place occupied by WS in this class is explained by the fact that WSs are driven by a natural reconnection‐like (tearing) instability, which is driven by the mutual attraction of the parallel current filaments. The non‐reconnection regime is explained by the initial magnetization of electrons (so that the dissipation is provided by the Landau resonance with unmagnetized ions (Schindler, 1974)) and the development of DFs in its nonlinear phase (e.g., Sitnov et al., 2013; Sitnov & Swisdak, 2011). Indeed, Events 2–6 developed in the DF trailing region.

To conclude, the concept of plasma WSs, introduced two decades ago (Lin & Swift, 2002) and recently reiterated on the fully kinetic level in 3‐D PIC simulations (Sitnov, Motoba, & Swisdak, 2021), offers an interesting alternative to the paradigm of divergent flows as a consequence of the magnetic topology change. In this study, using the high‐resolution MMS measurements, we have provided the characteristic fields and plasma variations of electron and ion WSs. The features we describe share many similarities with those predicted in simulations and help separate WSs from processes near the EDR and electron‐only reconnection regimes.

## Supporting information

Supporting Information S1

## Data Availability

The MMS data used in this study are publicly available at https://lasp.colorado.edu/mms/sdc/public/. The SMU and SML indices are available on the SuperMAG website (https://supermag.jhuapl.edu/). SuperMAG is an international collaboration with many organizations and institutes, and it is funded by National Science Foundation. The OMNI solar wind data are publicly available at https://omniweb.gsfc.nasa.gov. DM results are available at https://doi.org/10.5281/zenodo.6401960.
