# Supplementary material for: A New Perspective on Magnetotail Electron and Ion Divergent Flows: MMS Observations
Source: J Geophys Res Space Phys. 2022 Sep 30;127(10):e2022JA030514. doi: 10.1029/2022JA030514 (PMC9788156; doi:10.1029/2022JA030514)
Supplement: Supplementary file 1 — Supporting Information S1 [file JGRA-127-e2022JA030514-s001.pdf]

AGU PUBLICATIONS

Journal of Geophysical Research Space Physics

Supporting Information for

A New Perspective on Magnetotail Electron and Ion Divergent Flows: MMS Observations

T. Motoba[1], M. I. Sitnov[1], G. K. Stephens[1], and D. J. Gershman[2]

[1] The Johns Hopkins University Applied Physics Laboratory, Laurel, Maryland, USA, [2] NASA Goddard Space Flight Center, Greenbelt, MD, USA,

Contents of this file

Figures S1-S13

Additional Supporting Information (Files uploaded separately)

Introduction

Texts S1-S3 contain descriptions of three other watershed (WS) events, Events 4–6: Event 4 and Event 5 on 6 July 2018, and Event 6 on 19 August 2018. Figures S1-S8 are cited in the main text, while Figures S9-S13 are primarily cited in Texts S1-S3.

Text S1. 6 July 2018 Event: Events 4

Both Events 4 and 5 took place at 1140–1150 UT on 6 July 2018, during which the recovery phase of a small substorm was going on (Figure S9) and the MMS spacecraft were located at ~14 R_E downtail, (X, Y, Z) = (–13.2, 2.9, 3.6) R_E in GSM. As evident in the left and central panels of Figures 11a, their data mining (DM) reconstruction results indicate that the MMS consternation, being also located close to the global X-lines, is close to dipolarized regions with larger B_z values than those for Events 1–3.

Event 4, displayed in the left panels of Figures 11b–11g, occurred in the near-Earth tail CPS where $|B_z| > |B_x|$ (Figure 11b); plasma density ≥ 0.3 cm^{-3} (Figure 11e); plasma β > 1 (the left panel of Figure 11f); and unmagnetized ions ($Q_i^{1/2} > 0.1$, Figure 11g). During this interval, MMS 1 observed three successive DFs characterized by transient enhancements in B_z (Figure 11b) and corresponding sudden drops in plasma density (Figure 11e). The DFs occurred under earthward ion flow conditions with $V_{ix\perp}$ > 200 km s^{-1} (Figure 11c). After the DFs, $V_{ix\perp}$ experienced an earthward-to-tailward turning at ~1143:05 UT under stable positive B_z conditions (≥ 10 nT), which may be interpreted as an ion WS. In the enhanced $Q_i^{1/2}$ region, three distinct $V_{ex\perp}$ reversals were detected at or

behind the DFs (Figure 11d). Particularly, the first two of these electron WSs had large amplitudes of $|V_{ex\perp}| > 1{,}000$ km s^{-1}.

Figure S10 provides a zoomed-in view of Event 4 at 1142:20–1142:30 UT, including only the first and second electron WSs that occurred at ~1142:24.2 and ~1142:28.1 UT, respectively. Note that the data are shown in GSM coordinates. The field and plasma features are similar to those of most other electron WS events considered in this study. A unique feature is strong negative-to-positive spikes of E_y with $|E_y| \sim 30$ mV m^{-1} (Figure S10f) that were correlated with reversals of $V_{ex\perp}$ and $(\mathbf{E} \times \mathbf{B})_x/|B|^2$ (Figure S10d). This feature is consistent with tailward-earthward oscillations of flux tubes with magnetized electrons against the unmagnetized ion background.

Despite these similarities, the energy conversion features (Figure S10h) are different between the first and second electron WSs. In the first electron WS, the Joule heating rate $\mathbf{J}\cdot\mathbf{E}'$ was negative (~ –0.3 nW m^{-3}) but largely different from the energy conversion rate of positive $\mathbf{J}\cdot\mathbf{E}$ ~0.1 nW m^{-3}. In the second electron WS, on the other hand, both $\mathbf{J}\cdot\mathbf{E}'$ and $\mathbf{J}\cdot\mathbf{E}$ were positive but their amplitudes were different ($\mathbf{J}\cdot\mathbf{E}' \sim 0.2$ nW m^{-3} and $\mathbf{J}\cdot\mathbf{E} > 0.4$ nW m^{-3}).

The third electron WS at 1142:37.8 UT (not zoomed-in here) was also detected in the DF trailing region. It had a smaller amplitude ($|V_{ex\perp}| < 1000$ km s^{-1}) than the first and second electron WSs, but shared many other commonalities, including strong E_y reversals ($|E_y| > 10$ mV m^{-1}) correlated with $V_{ex\perp}$ and $(\mathbf{E} \times \mathbf{B})_x/|\mathrm{B}|^2$ reversals (not shown here).

Text S2. 6 July 2018 Event: Events 5

Events 5, displayed in the central panels of Figure 11, was detected approximately 6 minutes after Event 4. We consider it separately from Event 4 because the corresponding electron WS at 1148:37 UT was embedded in another ion WS at ~1148:45 UT. Similar to Event 4, the electron WS took place in a flux tube pileup region following a DF with $B_z > 20$ nT. Such large B_z values also explain relatively small values of the agyrotropy parameters, especially the ion parameter $Q_i^{1/2} < 0.1$.

Figure S11 presents a zoomed-in view of Event 5 at 1148:35–1148:45 UT. During the electron WS, the ion flow velocity was persistently positive but rather small ($V_{ix\perp} < 100$ km s^{-1}, Figure S11c), suggesting that the corresponding flux bundle was stopping and then rebounding at the time of the ion WS. In spite of relatively small values of the ion agyrotropy, electrons were decoupled from the convective ions. Furthermore, in contrast to ions, electrons closely followed the $\mathbf{E}\times\mathbf{B}$ drift motion (Figure S11b). It is interesting to note that this electron WS was not associated with any noticeable changes of the electron temperature and that the electron species was practically isotropic (Figure S11g). Yet, this electron WS was associated with substantial energy conversion and MHD heating (up to ~0.2 nW m^{-3}, Figure S11h), suggesting that it was an active region, although it unlikely drove any reconnection in that strongly dipolarized region.

Text S3. Events 6 on 19 August 2018

Event 6, displayed in the right panels of Figure 11, took place on 19 August 2018 when the MMS spacecraft were located at a downtail radial distance of ~17 R_E, (X, Y, Z) = (–16.9, 3.2, 3.9) R_E in GSM and the recovery phase of an isolated moderate substorm with a minimum SML of –375 nT was initiated (Figure S12). The DM reconstruction

indicates that the MMS spacecraft were very close to a strongly dipolarized region, as seen in Figure 11a. Indeed, it is evident from the right panels of Figure 11 that this WS was right at a DF. The *x* component of the perpendicular ion velocity, $V_{ix\perp}$ (Figure 11c), exhibited a flow reversal from earthward (> 1,000 km s^{-1}) to tailward (approximately –250 km s^{-1}) near 1759:00 UT under positive B_z field of ~10 nT (Figure 11b). This ion WS was preceded by an electron WS with both $V_{ex\perp}$ and $(\mathbf{E}\times\mathbf{B})_x/|B|^2$ reversals (Figure 11c). During the 3-min interval, the plasma density (Figure 11e) dropped at the DF but it remained relatively large (~0.4 cm^{-3}) with high β ~10 (Figure 11f). Similar to other WS events, this electron WS occurred in the magnetotail CPS region where ions were mostly unmagnetized with $Q_i^{1/2}$ ~0.2, whereas electrons were magnetized ($Q_e^{1/2}$ ~0.01 $\ll$ $Q_i^{1/2}$), as seen in Figure 11g.

Figure S13 presents a zoomed-in view of Event 6 at 1757:00–1757:10 UT. The most remarkable electron WS at ~1757:04.5 UT was accompanied by strong E_y reversals with the amplitude of ~20 mV m^{-1} (Figure S13f). It is interesting that, in spite of strong variations of the energy conversion and MHD heating rates exceeding 0.4 nW m^{-3} (Figure S13h), the electron temperature variations and anisotropy were less pronounced (Figure S13g). This electron WS resembles that of Events 4 and 5 with rapid tailward-then-earthward motions of the dipolarized flux tubes with magnetized electrons against the background of the earthward ion flows. Whereas these motions fit the formal definition of plasma WSs, they are likely caused by a mechanism different from the pre-reconnection processes described in Sitnov et al. (2021).

In summary, Events 4–6 share many commonalities with Events 1–3 mentioned in the main text. As mentioned in the main text, however, the most significant difference is that Events 4–6 occurred in the dipolarized tail regions with large B_z values (> 15 nT), in contrast to Events 1–3 that occurred in regions of relatively small B_z values (< 10 nT). Therefore, one possible mechanism explaining the plasma divergent flows for Events 4–6 is the ballooning/interchange instability in the vicinity of DFs.

References:

Gjerloev, J. W. (2012), The SuperMAG data processing technique, J. Geophys. Res., 117, doi:10.1029/2012JA017683.

Newell, P. T., and J. W. Gjerloev (2011), Evaluation of SuperMAG auroral electrojet indices as indicators of substorms and auroral power, J. Geophys. Res., 116, A12211, doi:10.1029/2011JA016779.

Sitnov, M. I., Stephens, G., Motoba, T., & Swisdak, M. (2021). Data mining reconstruction of magnetotail reconnection and implications for its first-principle modeling. Frontiers in Physiology, 9. https://doi.org/10.3389/fphy.2021.644884

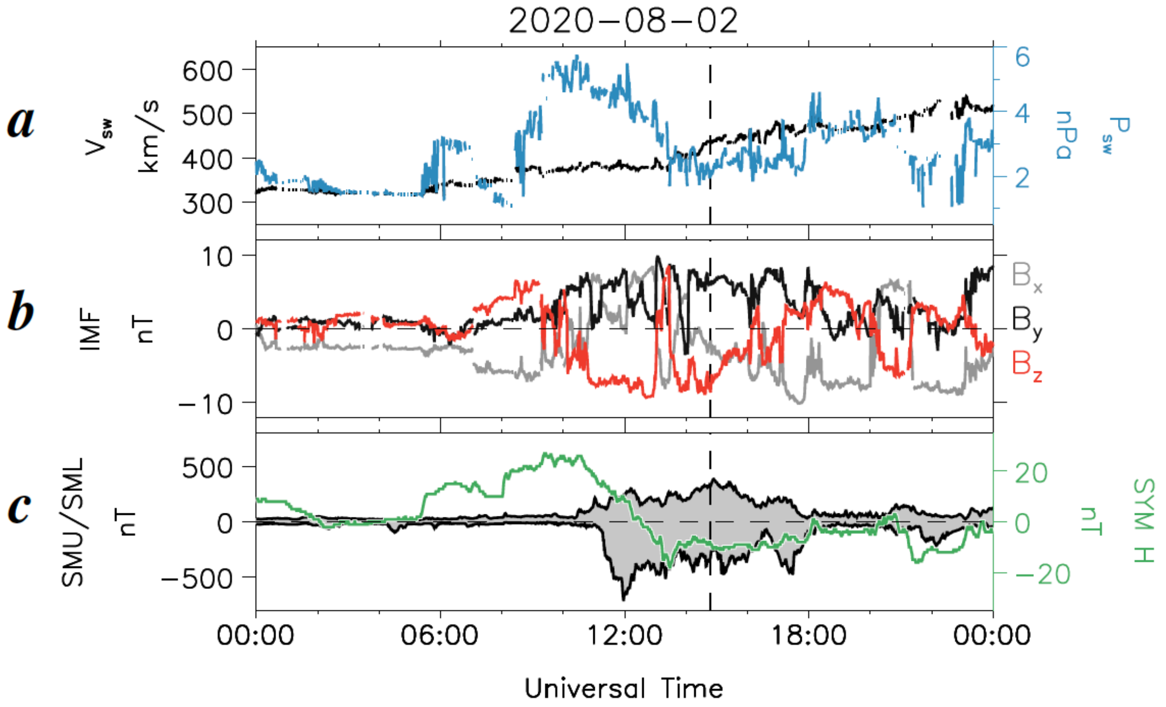

Figure S1. Solar wind and geomagnetic conditions on 2 August 2020. (a) Solar wind velocity (V_{sw}, black) and dynamic pressure (P_{sw}, blue). (b) Three components (B_x, gray; B_y, black; and B_z: red) of the interplanetary magnetic field (IMF) in GSM coordinates. (c) SuperMAG auroral electrojet indices, SMU and SML (analogous to AL and AU: Newell and Gjerloev, 2011; Gjerloev, 2012), and Sym H (green). Vertical dashed line denotes Event 1 at 1446:00–1448:30 UT.

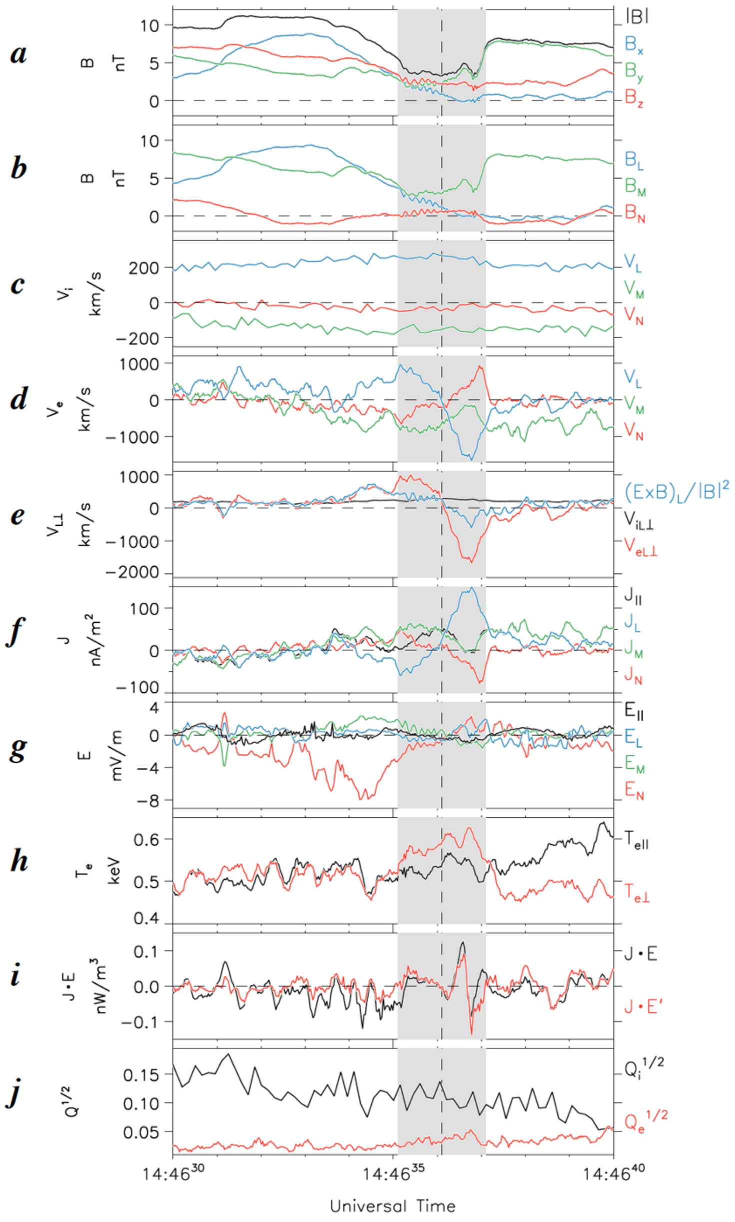

Figure S2. A zoomed-in plot of MMS 1 observations for Event 1 at 1446:30–1446:40 UT on 2 August 2020, but displayed in a local boundary (LMN) coordinate system. The LMN coordinate system was obtained by minimum variance analysis on the magnetic field for the 10-s interval (MVA-B): **L** (0.8528, –0.2474, 0.4599), **M** (0.0521, 0.9165, 0.3966), and **N** (–0.5196, –0.3143, 0.7945). Note that the LMN system is close to the GSM system. The MVA-B-derived **N** is consistent with the normal direction, (–0.5195, –0.4562, 0.7225) estimated from multi-spacecraft timing analysis on the B_x profile (see Figure S3a). The timing analysis also indicates that the neutral sheet moved along **N** with a speed of 51.2 km s^{-1}. (a) **B** in GSM (B_x, blue; B_y, green; B_z, red; and $|B|$, black). (b) **B** in LMN (B_L, blue; B_M, green; B_N, red). (c) $\mathbf{V}_\mathrm{i}$ in LMN (V_{iL}, blue; V_{iM}, green; and V_{iN}, red). (d) $\mathbf{V}_\mathrm{e}$ in LMN (V_{eL}, blue; V_{eM}, green, and V_{eN}: red). (e) $\mathbf{V}_{eL\perp}$ (red), $\mathbf{V}_{iL\perp}$ (black) and $(\mathbf{E}\times\mathbf{B})_\mathrm{L}/|B|^2$ (blue) in LMN. (f) **J** in LMN (J_L, blue; J_M, green; J_N, red; and $J_{||}$, black). (g) **E** in LMN (E_L, blue; E_M, green; E_N, red; and $E_{||}$, black). (h) $T_{e\perp}$ (red) and $\mathrm{T}_{\mathrm{e}||}$ (black). (i) $\boldsymbol{J}\cdot\boldsymbol{E}$ and $\boldsymbol{J}\cdot\boldsymbol{E}'$. (j) ion and electron agyrotropies, $Q_i^{1/2}$ (black) and $Q_e^{1/2}$ (red).

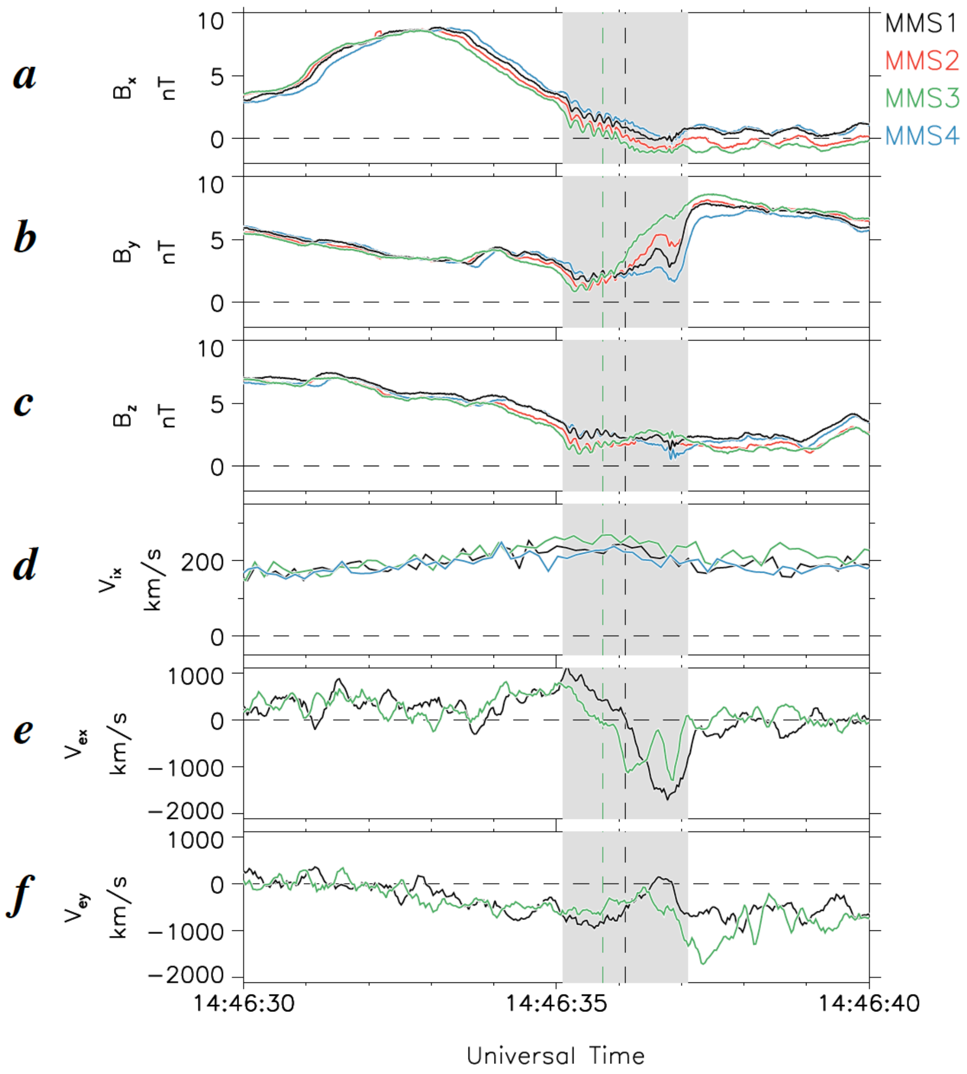

Figure S3. Four MMS observations around the electron WS for Event 1 at 1446:30–1446:40 UT on 2 August 2020. Shown from the top to bottom panels are (a) B_x; (b) B_y; (c) B_z; (d) V_{ix}; (e) V_{ex}; and (f) V_{ey} in GSM coordinates. Note that the FPI electron and ion moment data at MMS 2 and the FPI electron moment data at MMS 4 are not available. Both magnetic field and flow data show similar profiles among the different satellites, except for the time delay. A clear difference in the time of the neutral sheet (B_x = 0 nT) crossing/touching is present among the four spacecraft (panel a), first being observed by MMS 3 followed by MMS 2 and MMS 1, and then finally MMS4. The time delay is consistent with the difference between the V_{ex} reversal times at MMS 1 and MMS 3, denoted by black and green vertical dashed lines, respectively.

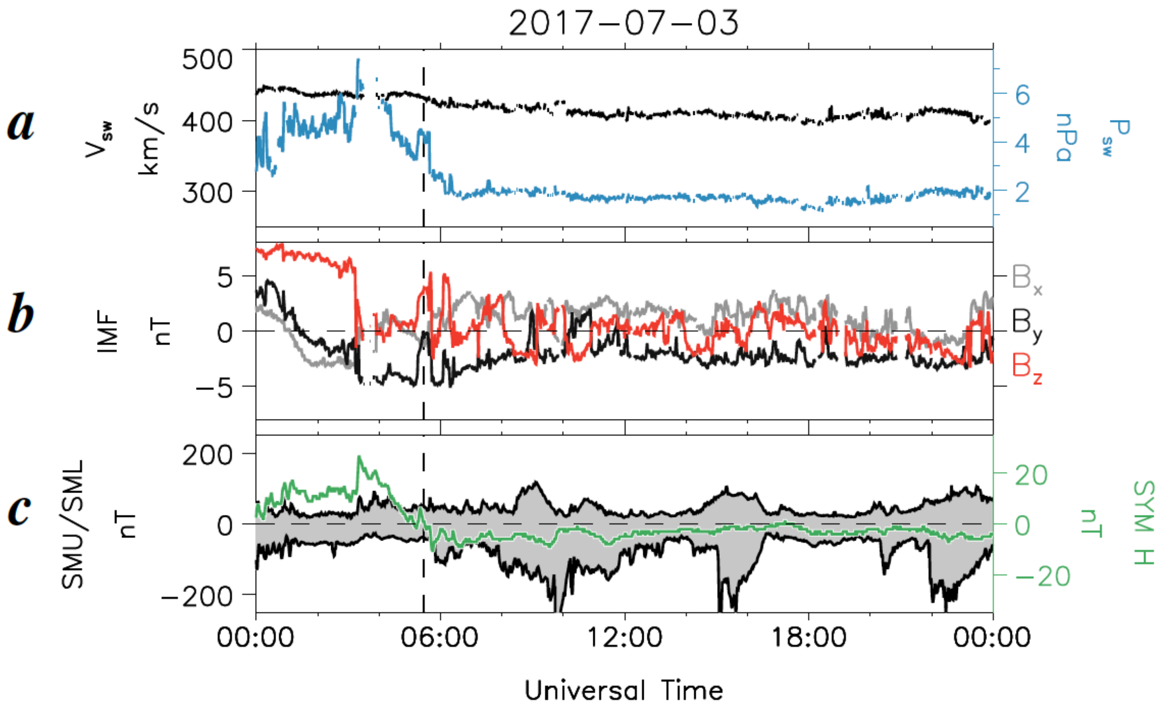

Figure S4. Same format as Figure S1 but for Event 2 on 3 July 2017. Vertical dashed line denotes Event 2 at 0526:30–0527:15 UT.

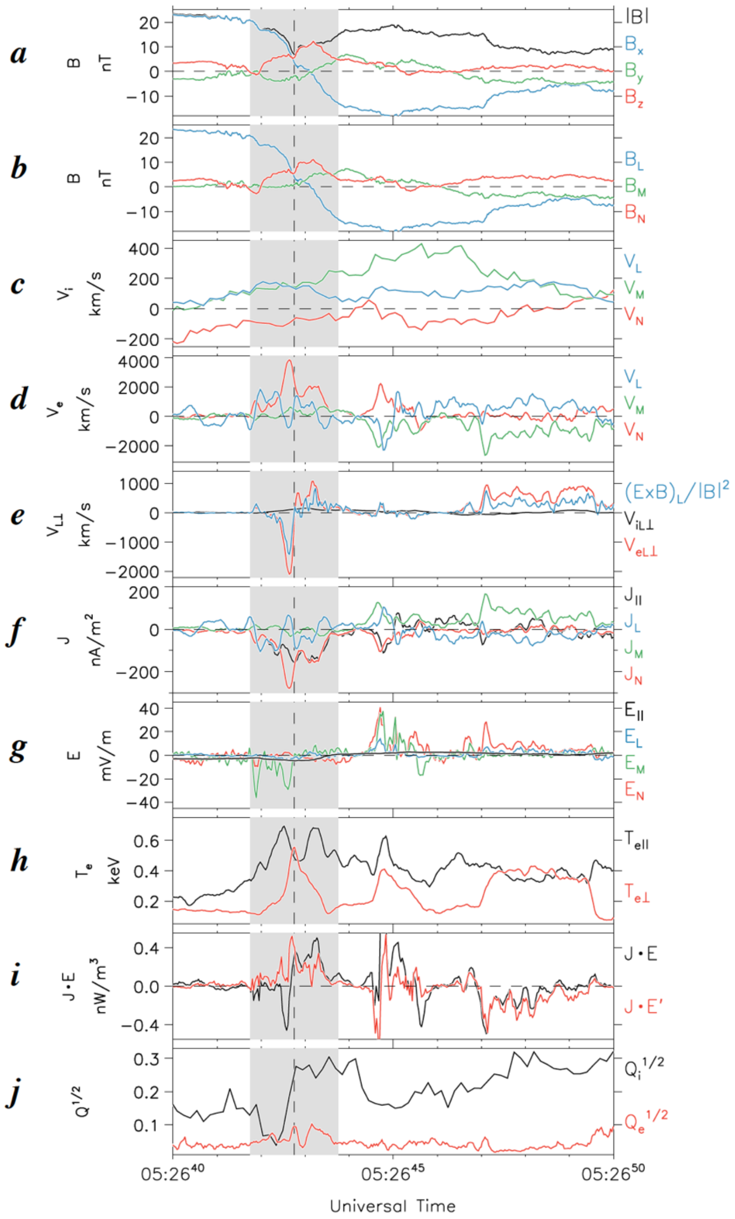

Figure S5. Same as Figure S2, but for Event 2 at 0526:40–0526:50 UT on 3 July 2017. The LMN coordinate system was obtained by MVA-B for the 10-s interval: **L** (0.9946, –0.0941, 0.0447), **M** (0.0667, 0.9046, 0.4210), and **N** (–0.0801, – 0.4158, 0.9059). The LMN system is close to the GSM system.

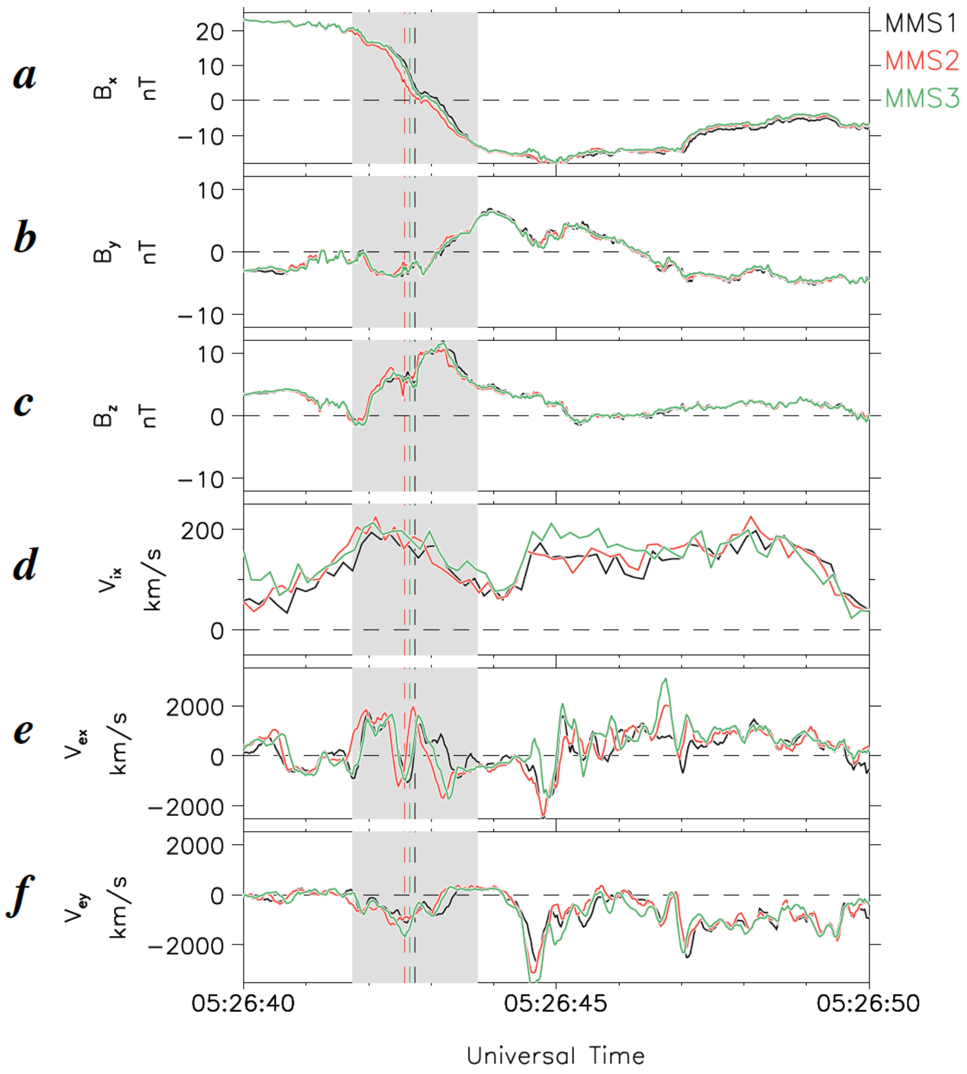

Figure S6. Same as Figure S3, but for Event 2 at 0526:40–0526:50 UT on 3 July 2017. Note that not all data from MMS 4 instruments are available for Event 2. A small difference in the time of the neutral sheet (B_x = 0 nT) crossing is present among the three spacecraft, first being observed by MMS 2 followed by MMS 3, and then finally MMS1, which is consistent with the time delays of the V_{ex} reversals among the three MMS probes.

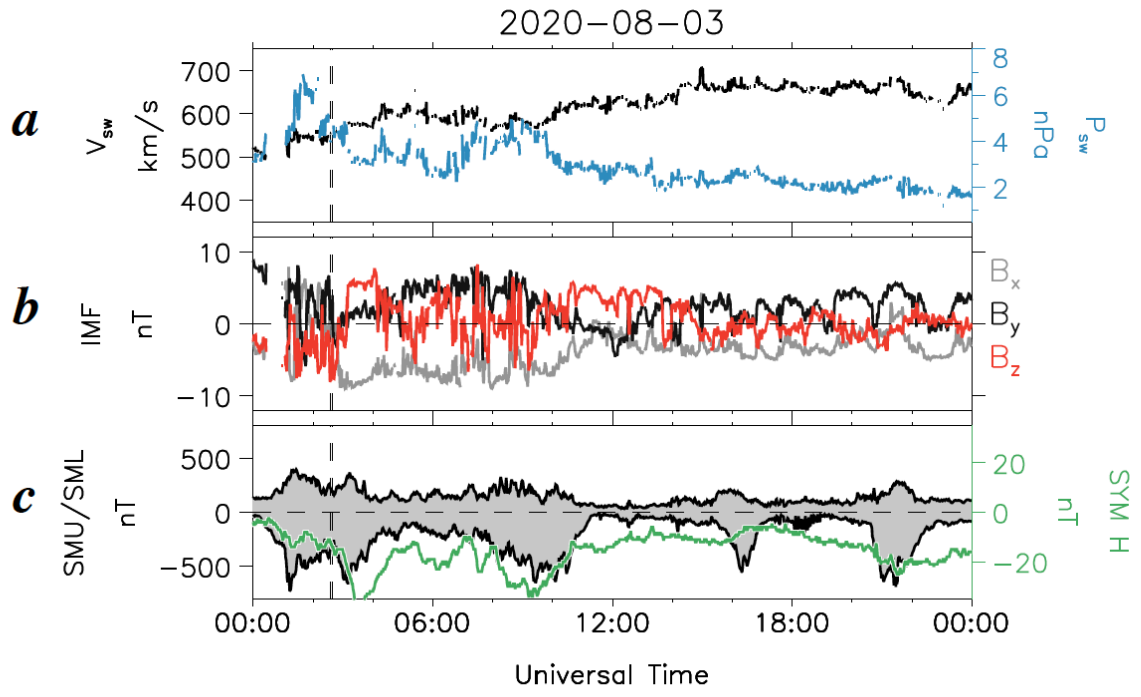

Figure S7. Same format as Figure S1 but for Event 3 on 3 August 2020. Vertical dashed line denotes Event 3 at 0234:15–0237:45 UT.

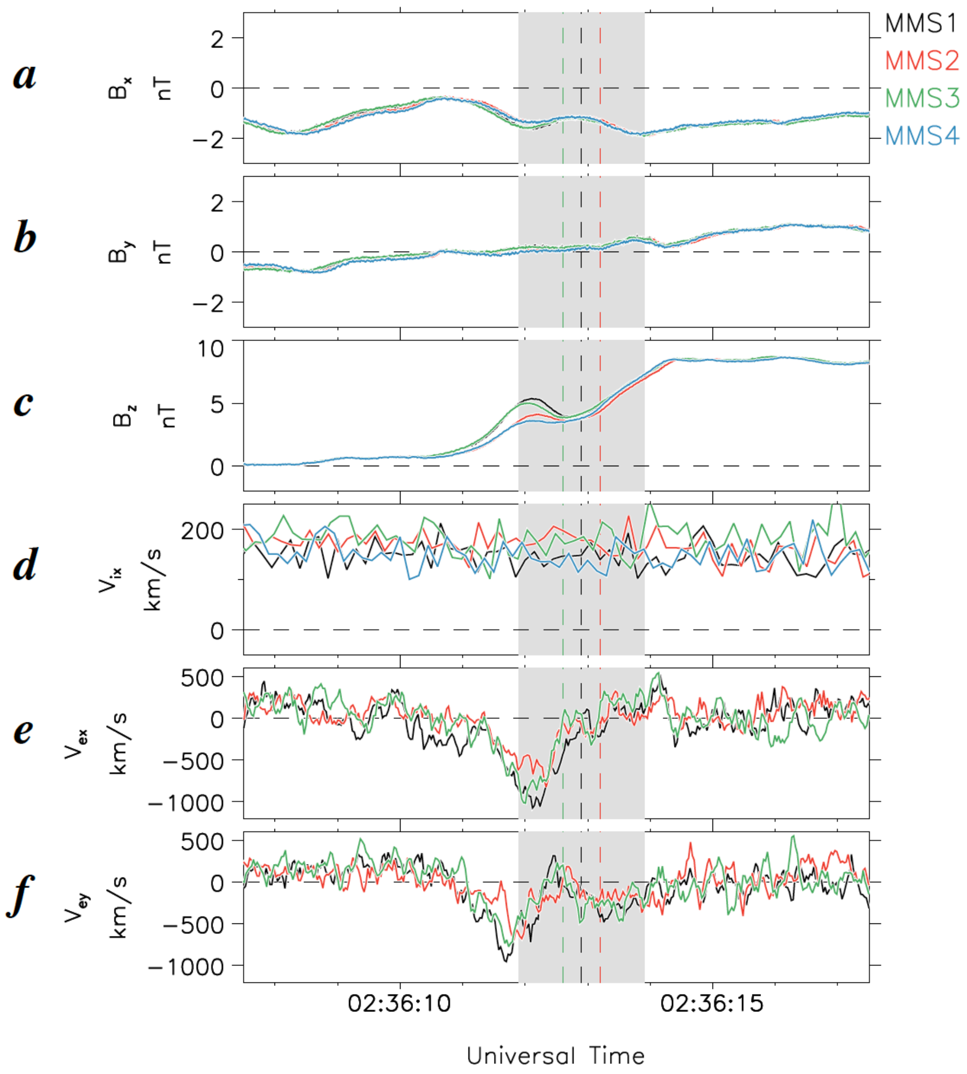

Figure S8. Same as Figure S3, but for Event 3 at 0236:07.5–0236:17.5 UT on 3 August 2020. Note that the FPI electron moment data at MMS 4 are not available throughout the interval. A difference in the time of the B_z enhancement is obvious among the spacecraft, which is consistent with the difference in the time of the V_{ex} reversal.

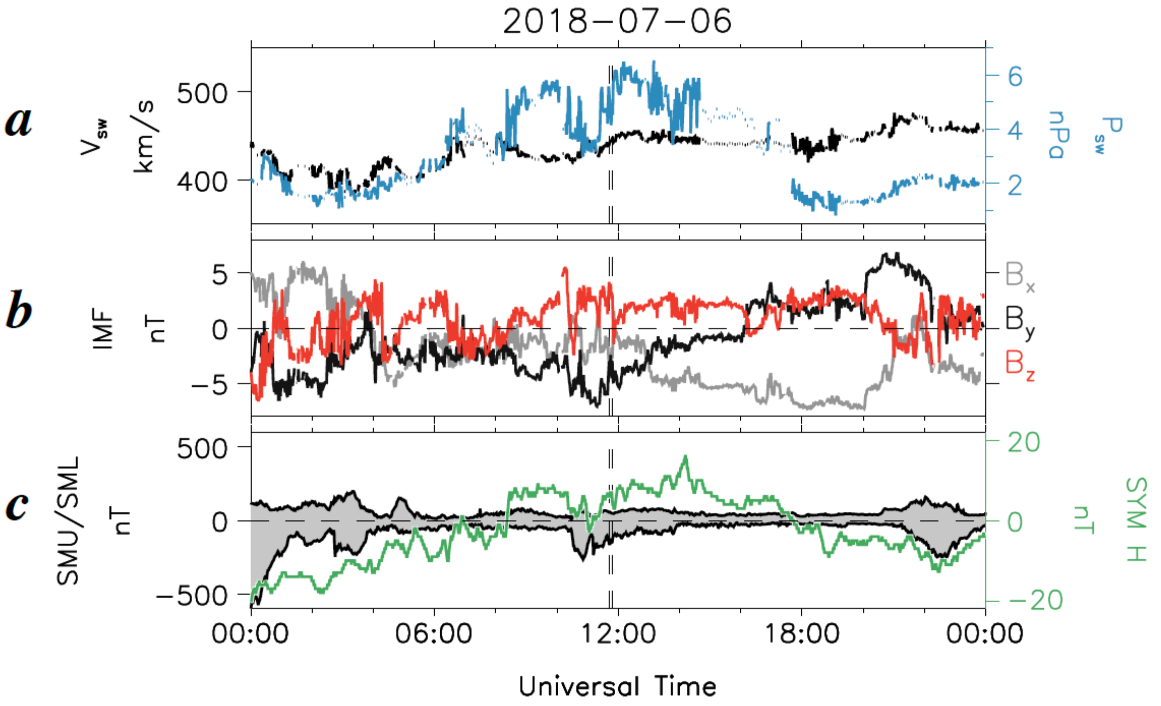

Figure S9. Same format as Figure S1 but for Events 4 and 5 on 6 July 2018. Two vertical dashed lines denote Event 4 at 1142:15–1143:15 UT and Event 5 at 1148:15–1149:15 UT, respectively.

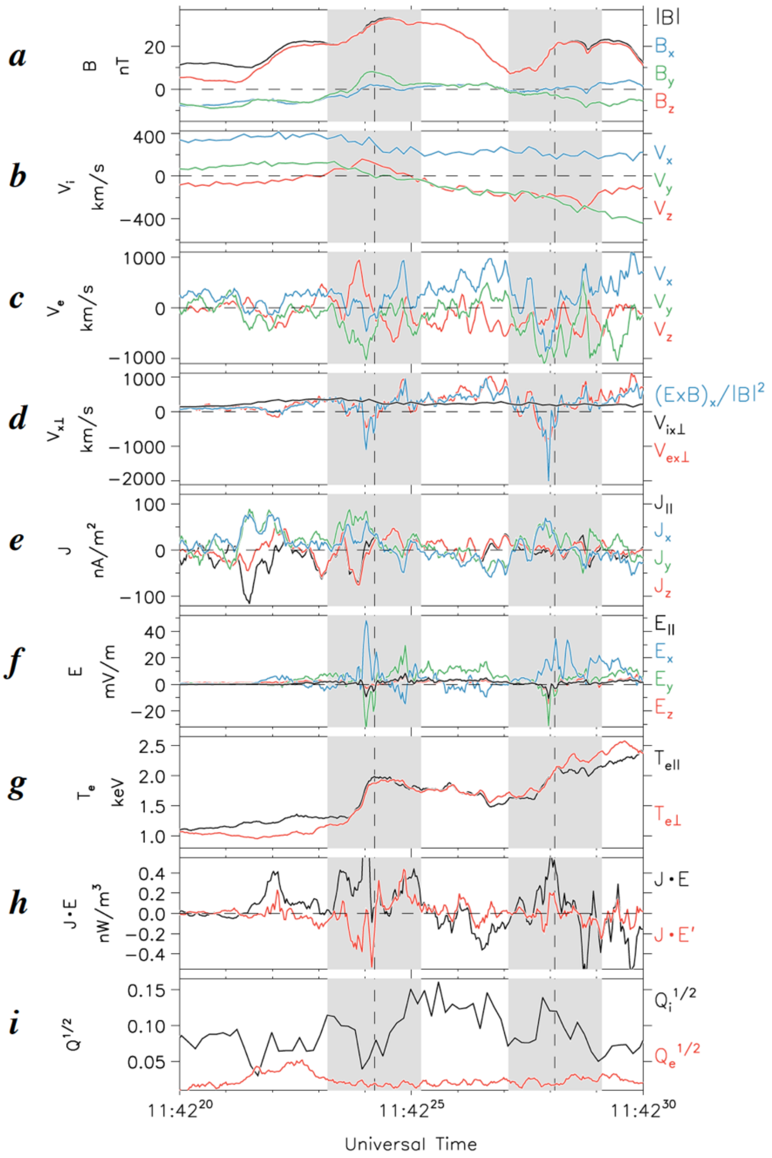

Figure S10. Zoomed-in plots of MMS 1 observations of Event 4 for a 10-s interval of 1142:20–1142:30 UT on 6 July 2018, which appears between two dashed lines in the left panels of Figure 11. All the parameters are presented in the GSM coordinate system. During this interval the first and second electron WSs occurred at ~1142:24.2 and ~1142:28.1 UT, respectively (denoted by dashed lines along with gray shadings). (a) Magnetic field (B_x, blue; B_y, green; B_z, red; and $|B|$, black). (b) Ion velocity (V_{ix}, blue; V_{iy}, green; and V_{iz}, red). (c) Electron velocity (V_{ex}, blue; V_{ey}, green; and V_{ez}, red). (d) $V_{ex\perp}$ (red), $V_{ix\perp}$ (black), and $(\mathbf{E}\times\mathbf{B})_x/|B|^2$ (blue). (e) Current density (J_x, blue; J_y, green; J_z, red; and $J_{||}$, black). (f) Electric field (E_x, blue; E_y, green; E_z, red; and $E_{||}$, black). (g) $T_{e\perp}$ (red) and $T_{e||}$ (black). (h) $\boldsymbol{J}\cdot\boldsymbol{E}$ (black) and $\boldsymbol{J}\cdot\boldsymbol{E_e}'$ (red). (i) ion and electron agyrotropies, $Q_i^{1/2}$ (black) and $Q_e^{1/2}$ (red).

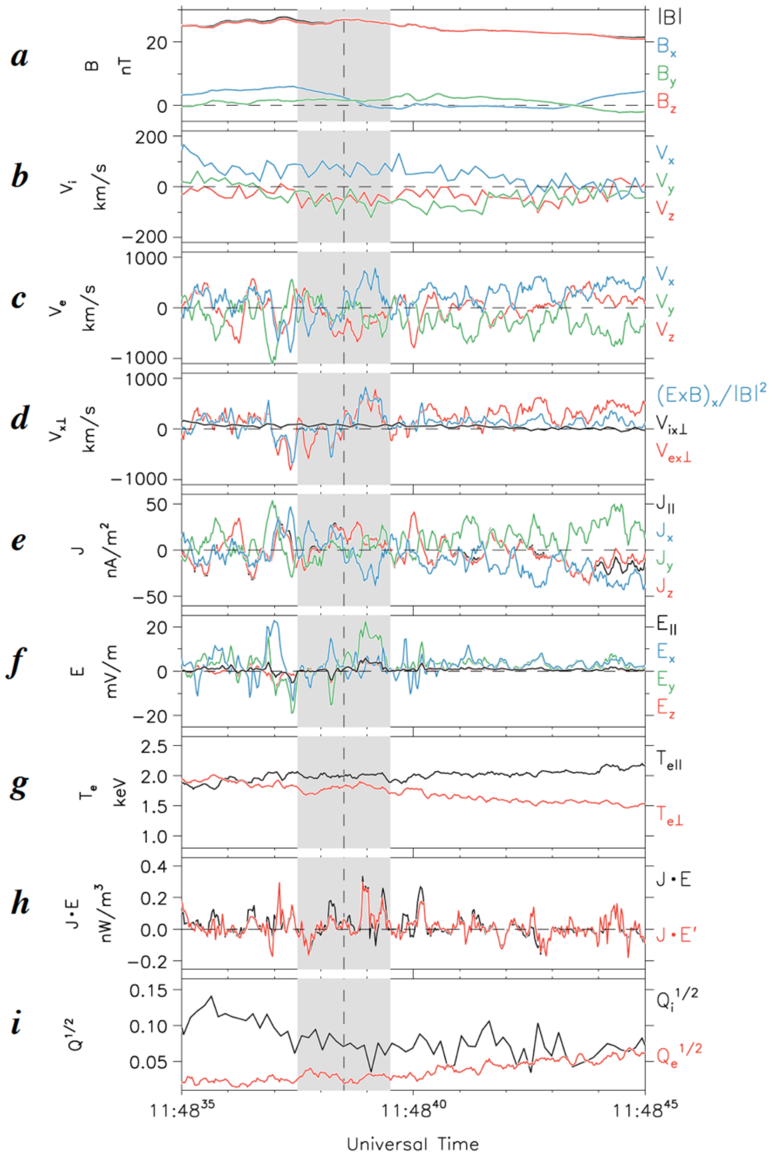

Figure S11. Same as Figure S10 but for Event 5 at 1148:35–1148:45 UT on 6 July 2018, which appears between two dashed lines in the central panels of Figure 11. Dashed line denotes the electron WS at 1148:38.5 UT.

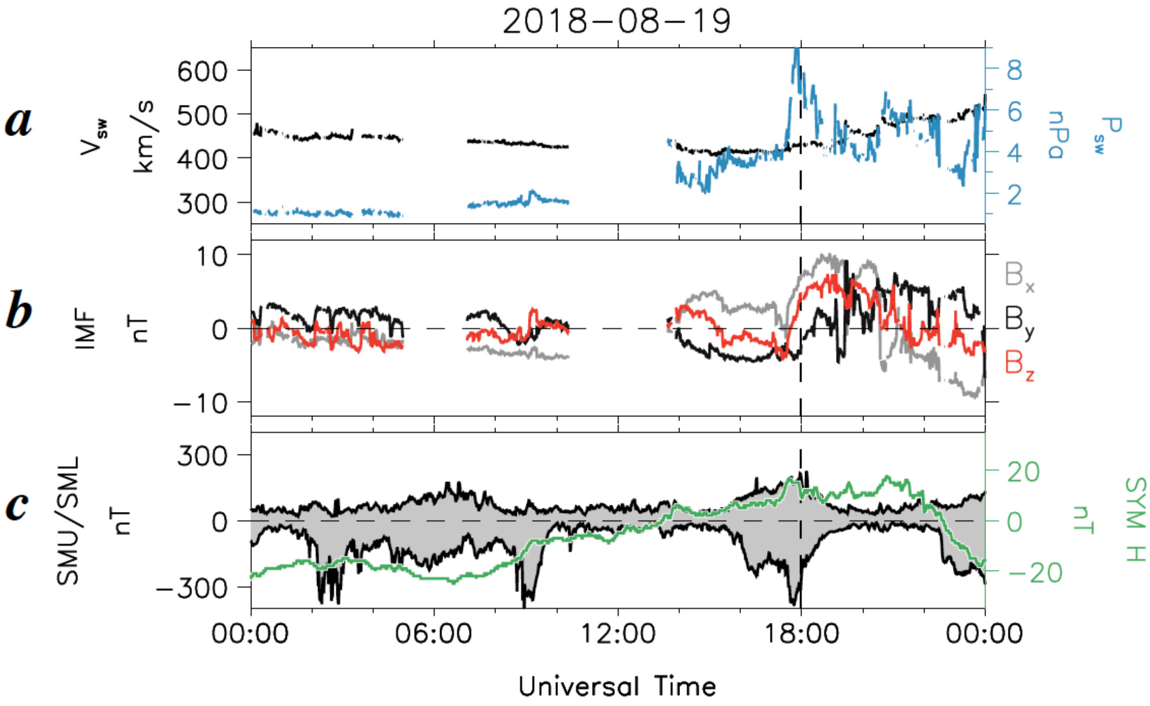

Figure S12. Same format as Figure S1 but for Event 6 on 19 August 2018. Vertical dashed line denotes Event 6 at 1756:30–1759:30 UT.

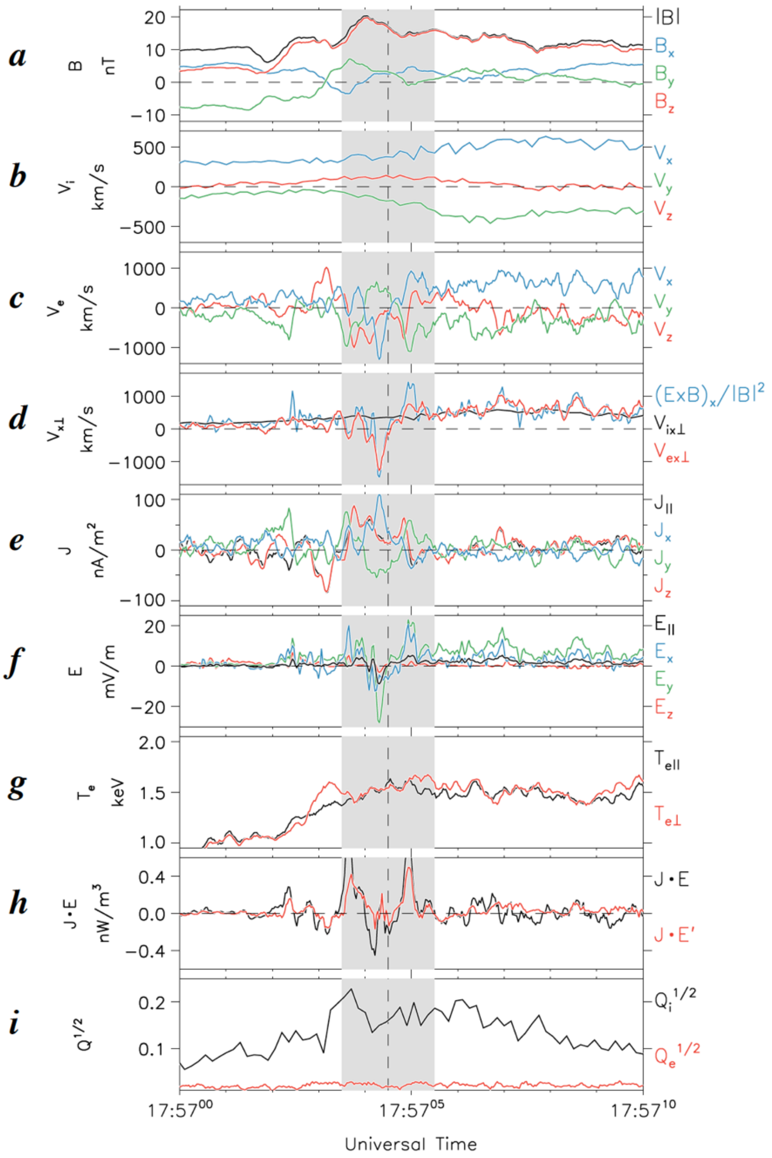

Figure S13. Same format as Figure S10 but for Event 6 at 1757:00–1757:10 UT on 19 August 2018, which appears between two dashed lines in the right panels of Figure 11. Dashed line indicates the electron WS at 1757:04.5 UT.
